# New label‐free automated survival assays reveal unexpected stress resistance patterns during *C. elegans* aging

**DOI:** 10.1111/acel.12998

**Published:** 2019-07-16

**Authors:** Alexandre Benedetto, Timothée Bambade, Catherine Au, Jennifer M.A. Tullet, Jennifer Monkhouse, Hairuo Dang, Kalina Cetnar, Brian Chan, Filipe Cabreiro, David Gems

**Affiliations:** ^1^ Department of Genetics, Evolution and Environment, Institute of Healthy Ageing University College London London UK; ^2^ Division of Biomedical and Life Sciences Lancaster University Lancaster UK; ^3^ School of Biosciences University of Kent Canterbury UK; ^4^ Division of Infection, Immunity & Respiratory Medicine University of Manchester Manchester UK; ^5^ MRC London Institute of Medical Sciences, Imperial College London London UK

**Keywords:** aging, autophagy, *C. elegans*, infection, stress, survival

## Abstract

*Caenorhabditis elegans* is an excellent model for high‐throughput experimental approaches but lacks an automated means to pinpoint time of death during survival assays over a short time frame, that is, easy to implement, highly scalable, robust, and versatile. Here, we describe an automated, label‐free, high‐throughput method using death‐associated fluorescence to monitor nematode population survival (dubbed LFASS for label‐free automated survival scoring), which we apply to severe stress and infection resistance assays. We demonstrate its use to define correlations between age, longevity, and severe stress resistance, and its applicability to parasitic nematodes. The use of LFASS to assess the effects of aging on susceptibility to severe stress revealed an unexpected increase in stress resistance with advancing age, which was largely autophagy‐dependent. Correlation analysis further revealed that while severe thermal stress resistance positively correlates with lifespan, severe oxidative stress resistance does not. This supports the view that temperature‐sensitive protein‐handling processes more than redox homeostasis underpin aging in *C. elegans*. That the ages of peak resistance to infection, severe oxidative stress, heat shock, and milder stressors differ markedly suggests that stress resistance and health span do not show a simple correspondence in *C. elegans*.

## 1 INTRODUCTION

Survival assays are widely performed in both basic and applied biomedical research to assess the frailty of organisms, tissues, and cells, and to determine the toxicity or efficacy of a chemical agent. In such assays, death is usually revealed by a light signal and/or an enzymatic reaction, requiring reagents that may interfere with the process under study (Atale, Gupta, Yadav, & Rani, 2014; Gill, Olsen, Sampayo, & Lithgow, 2003). The nematode *C. elegans* is a widely used experimental model in basic, pharmacological, and environmental research (Leung et al., 2008), and for the study of parasitic worm species (Holden‐Dye & Walker, 2007; Jones, Buckingham, & Sattelle, 2005) that affect crops, cattle, and 3.5 billion people (Ojha, Jaide, Jinawath, Rotjanapan, & Baral, 2014). Its small size, optical transparency, and good genetics make *C. elegans* a convenient model organism for high‐throughput chemical and bacterial library screens for the development of antihelminthic and antiaging drugs, and for elucidating the biology of host–pathogen interactions (Marsh & May, 2012). Since the late 1990s, *C. elegans* screening platforms have evolved to include microfluidics and automated robotic arms (Crane, Chung, & Lu, 2009; Rajamuthiah et al., 2014). Yet, full automation of *C. elegans* survival assays has been limited by death scoring techniques. This is very relevant to the aging field, where daily manual monitoring of worms for lifespan measurements had limited the throughput of aging studies. Recent techniques have enabled tracking of worm behavior over their lifespan, allowing for automated lifespan measurements (Churgin et al., 2017; Crane et al., 2009; Park, Jung, & Lee, 2017; Stroustrup et al., 2013). They include the Lifespan Machine that uses a scanner bed and can accommodate tens of worm plates at once (Stroustrup et al., 2013), and the WorMotel that uses 48‐well silicon chips to image arrays of singled freely moving nematodes (Churgin et al., 2017). However, many stress survival assays primarily aim to measure median time of death, and what is particularly needed for their improvement is higher throughput and easier implementation, rather than the breadth of behavioral and imaging data afforded by other automated approaches. Moreover, determining time of death based on cessation of movement as it is traditionally done in lifespan assays (Churgin et al., 2017; Park et al., 2017; Stroustrup et al., 2013; Sutphin & Kaeberlein, 2009) may not be accurate enough for shorter‐term assays (Coburn et al., 2013; Galimov et al., 2018) and can be confounded by genetic background (e.g., *unc‐22*), experimental conditions (e.g., levamisole treatment, Figure S2), and old age (Podshivalova, Kerr, & Kenyon, 2017), where nematode mobility is greatly reduced.

We previously discovered that an endogenous burst of blue fluorescence, dubbed *death fluorescence* (DF), generated by autofluorescence de‐quenching of anthranilic acid conjugates, occurs in nematodes’ intestine at the onset of organismal death (Coburn et al., 2013; Coburn & Gems, 2013; Galimov et al., 2018). Anthranilates are tryptophan‐derived compounds generated by action of the kynurenine pathway (Coburn et al., 2013). We have exploited this natural phenomenon to develop automated, hassle‐free, and label‐free *C. elegans* survival assays for high‐throughput stress‐ and infection‐sensitivity screens, using standard microplate readers and a simple, newly created autofluorescence data analysis program.

## 2 RESULTS

### 2.1 Time‐lapse recording of *C. elegans* death fluorescence (DF) allows for automated, rapid, and sensitive multiplexed survival assays

**Figure 1:**
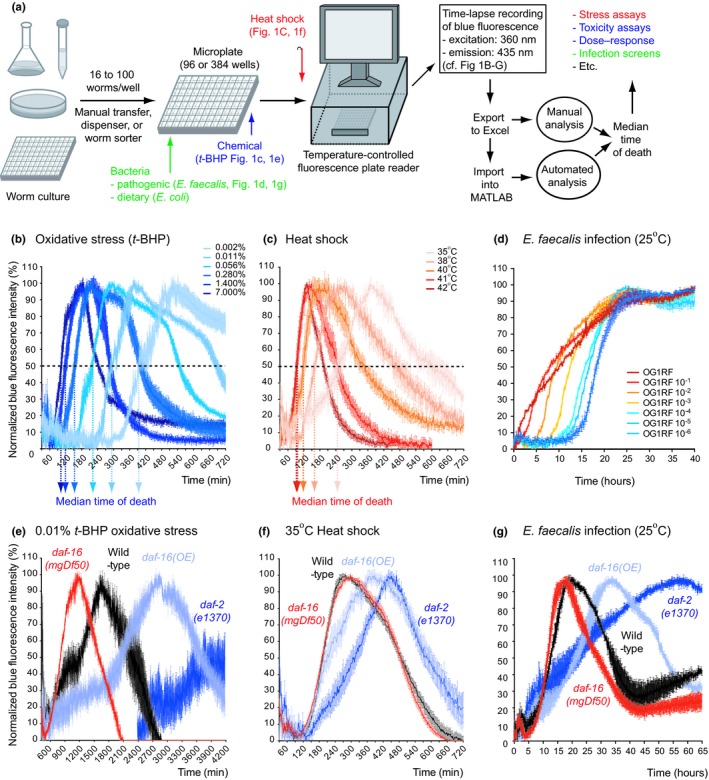
LFASS provides robust automated scoring and analysis of *C. elegans* survival assays. (a) The LFASS pipeline. Dose dependency of *t*‐BHP‐induced oxidative stress (b), thermal stress (c), and *E. faecalis* bacterial infection (d) resistance measured by LFASS. Automated measurement of resistance to oxidative stress (e), thermal stress (f), and *E. faecalis* bacterial infection (g) by LFASS discriminates between infection sensitive and resistant IIS mutants

From time‐lapse recordings of blue fluorescence during killing assays, we first verified that the timing of DF events in a population follows a Poisson distribution (Figure S1), and approximates a normal distribution when the median time of death exceeds 30 min (Figure S1b). Considering the total fluorescence of a nematode population over time, we found that its half‐maximal fluorescence corresponds to half the worms undergoing DF (Figure S1a,b). Hence, the time of half‐maximal fluorescence corresponds to the median time of death, which is the key parameter sought in survival assays. This prompted us to develop new, label‐free, high‐throughput, and automated survival assays relying on death fluorescence. To enable multiple conditions to be tested in parallel and to limit phototoxicity, we opted for a 384‐well microplate format to be read by a narrow bandpass fluorescence plate reader (Figure 1a). DF was optimally recorded with excitation/emission wavelengths of 360/435 nm (Figure S1c) using as few as 16 worms per well. With this setup, each measurement took 0.8 s, allowing 384 samples to be measured in under 5 min.

To assess the sensitivity of the method, we tested whether it could discriminate between different stress levels. Wild‐type adult hermaphrodites exposed to oxidative stress (0.002%–7% by volume *tert*‐butyl hydroperoxide [*t*‐BHP]) or heat stress (35–42°C) died within 8 hr, while those exposed to the pathogenic bacterium *Enterococcus faecalis* died within 24 hr. A robust dose dependency was observed for susceptibility to oxidative stress (Figure 1b), heat stress (Figure 1c), and infection **(**Figure 1d**)**. All three stress assays also clearly discriminated between strains with altered insulin/IGF‐1 signaling (IIS): a *daf‐16(mgDf50)* (FoxO) null mutant (stress sensitive), a *daf‐16* overexpresser, and a *daf‐2* insulin/IGF‐1 receptor reduction in function mutant (both stress resistant) (Figure 1e‐g). This is largely consistent with previous findings (Garsin et al., 2003; Henderson & Johnson, 2001; Lithgow, White, Melov, & Johnson, 1995; Tullet et al., 2017, 2008), though the hypersensitivity of *daf‐16 (mgDf50)* to death from *E. faecalis* infection was not detected using previous methods (Garsin et al., 2003; Zou, Tu, Niu, Ji, & Zhang, 2013). Hence, this label‐free automated survival scoring method (henceforth referred to as LFASS) proved sensitive, robust, and accurate in a variety of assays, indicating its suitability for high‐throughput approaches.

### 2.2 LFASS reveals a rise and fall in severe stress resistance during *C.* *elegans* aging

To further test its functionality, we used LFASS to re‐examine links between stress resistance and aging, which is tedious to study by traditional means. A long‐standing view is that stochastic damage accumulation contributes to aging and that mechanisms that protect against such damage increase both stress resistance and lifespan (Harman, 1956; Lithgow & Walker, 2002; Shore & Ruvkun, 2013). Hence, accumulation of damage and loss of maintenance mechanisms should lead to a decline in stress resistance with advancing age. Consistent with this, aging *C. elegans* have shown increased susceptibility to a range of moderate stresses (i.e., stresses that take several days to cause death) (Darr & Fridovich, 1995; Labbadia & Morimoto, 2015; Vanfleteren, 1993; Youngman, Rogers, & Kim, 2011). Perhaps due to the technical difficulty of gathering data from multiple samples at short time intervals, the sensitivity of aging nematodes to more severe stress has not been systematically assessed.

**Figure 2:**
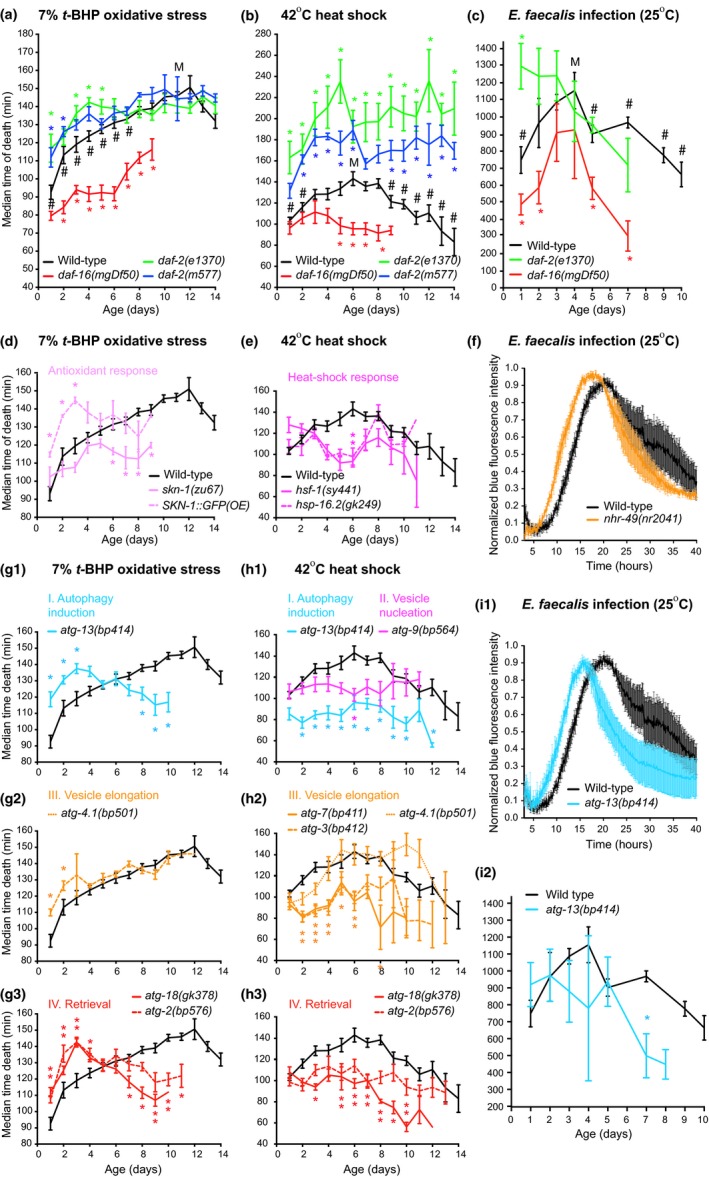
LFASS reveals distinct autophagy‐dependent patterns of stress resistance to severe oxidative stress (a, d, g), heat shock (b, e, h), and *E. faecalis* infection (d, f, i) in aging *C. elegans* IIS mutants. (a) Severe oxidative stress resistance in WT hermaphrodites increases until day 10 in a DAF‐16‐independent manner, reaching *daf‐2* resistance levels. (b) Severe thermal stress resistance increases until day 6 in a DAF‐16‐dependent manner in WT hermaphrodites before decreasing with age, but never reaches *daf‐2* resistance levels. (c) Severe *E. faecalis* infection resistance peaks at day 4 in WT and *daf‐16* and at day 1 in *daf‐2* hermaphrodites. (d) Severe oxidative stress resistance requires SKN‐1. (e) Severe thermal stress resistance involves HSF‐1 and heat‐shock proteins. (f) Severe *E. faecalis* infection resistance involves NHR‐49. (g, h, i) Autophagy is required for WT patterns of severe oxidative, thermal, and infection (*E. faecalis*) stress resistance with age. Error bars, *SEM*. M: peak resistance for wild‐type. Comparison to age‐matched wild‐type: * *p* < 0.05 down to *p* < 0.0001. Comparison to M within wild‐type values # *p* < 0.05 down to *p* < 0.0001. Animals were aged at 25˚C, and day 0 marks the late L4 stage

We therefore used the high temporal resolution capacity of LFASS to examine the effects of severe stress on *C. elegans* survival as they age. Specifically, we exposed adult worms to 7% *t*‐BHP (by volume, i.e., 777 mM, as opposed to 5 or 7.5 mM, as used previously) (Tullet et al., 2017, 2008) and 42°C (as opposed to 35°C as previously) (Labbadia & Morimoto, 2015), which in young adults both give a median survival time of only 1.5 hr (Figure 2a,b). We also studied how age affects resistance to accelerated *E. faecalis* OG1RF infection (death within 36 hr instead of 3 days) as a third severe stress paradigm. This led to some unexpected findings. Wild‐type nematodes showed initial increases in resistance to severe oxidative stress up to day 10 of adulthood, to high thermal stress up to day 6, and to severe *E. faecalis* infection up to day 4 (Figure 2a‐c), before exhibiting age‐dependent declines in stress resistance. It is unlikely that early increases in stress resistance are due to an age‐associated decrease in ingestion of *t*‐BHP or *E. faecalis* since this effect persisted in animals with genetically or pharmacologically impaired feeding (Figures S2d and S4a,b) and was susceptible to mutational suppression (Figure 2d‐i). These results imply that young adult *C. elegans* differ markedly in terms of their susceptibility and resistance to moderate versus severe stressors.

### 2.3 Rising severe stress resistance in early adulthood shows differential DAF‐16 dependence

To gain insight into the genetic specification of wild‐type severe stress resistance profiles, we next studied several well‐characterized age (altered lifespan) mutants, monitoring severe stress resistance throughout life, which the high‐throughput capability of LFASS makes possible. The IIS pathway is conserved from invertebrates to mammals, regulating growth, metabolism, and aging (Hesp, Smant, & Kammenga, 2015). Mutations affecting the *daf‐2* IIS receptor extend lifespan and increase stress resistance, while those affecting the downstream transcription factor DAF‐16/FoxO shorten lifespan and cause stress hypersensitivity (Murphy & Hu, 2013). As expected, aging cohorts of *daf‐2* and *daf‐16* mutants showed resistance and hypersensitivity, respectively, to severe stress (Figure 2a,c, Table S1). Furthermore, the age increase in resistance to heat shock but not to *t*‐BHP or *E. faecalis* was largely *daf‐16*‐dependent (Figure 2a‐c, Tables S2–S4). This implies that age changes in *C. elegans* stress defense mechanisms differ between stress modalities.

### 2.4 Autophagy underpins stress resistance dynamics in a context‐dependent fashion

To further explore the genetic basis of wild‐type stress resistance profiles, we tested the effects of additional mutations known to influence stress resistance. We found that the *skn‐1* (*NRF2*) antioxidant transcription factor (An & Blackwell, 2003) was required for the early age increase in oxidative stress resistance, the *hsf‐1* heat‐shock transcription factor (Garigan et al., 2002) for thermal stress resistance, and the *nhr‐49* transcription factor (Van Gilst, Hadjivassiliou, Jolly, & Yamamoto, 2005) for *E. faecalis* resistance (Figure 2d‐f), consistent with earlier studies involving corresponding moderate stress paradigms (Sim & Hibberd, 2016; Vihervaara & Sistonen, 2014; Zhang, Davies, & Forman, 2015).

We then explored the role of autophagy in stress resistance profiles using mutations in *atg* genes required for autophagic function. In most contexts, *atg* mutants showed increased susceptibility to stress (Figure 2g‐i, Table S1), apart from the *atg4.1(bp501)* mutant which lacks one of the two partially redundant *C. elegans* homologs of ATG4 (Wu, Li, Wang, Noda, & Zhang, 2012), consistent with the documented role of autophagy in protection against stress (Chapin, Okada, Merz, & Miller, 2015; Mizushima, Levine, Cuervo, & Klionsky, 2008). Yet, it is worth noting that *atg* mutants (including *atg‐4.1*) showed enhanced resistance to oxidative stress for the first 3–4 days of adulthood (Figure 2g1‐g3). This underscores the Janus‐face nature of autophagy that can decrease or increase stress sensitivity and disease depending on stress modality and disease etiology (Benedetto & Gems, 2019; Shintani & Klionsky, 2004). Importantly, our results imply that the broad age‐dependent increase in severe stress resistance is promoted by autophagy.

### 2.5 Severe thermal but not oxidative stress resistance correlates well with longevity

**Figure 3:**
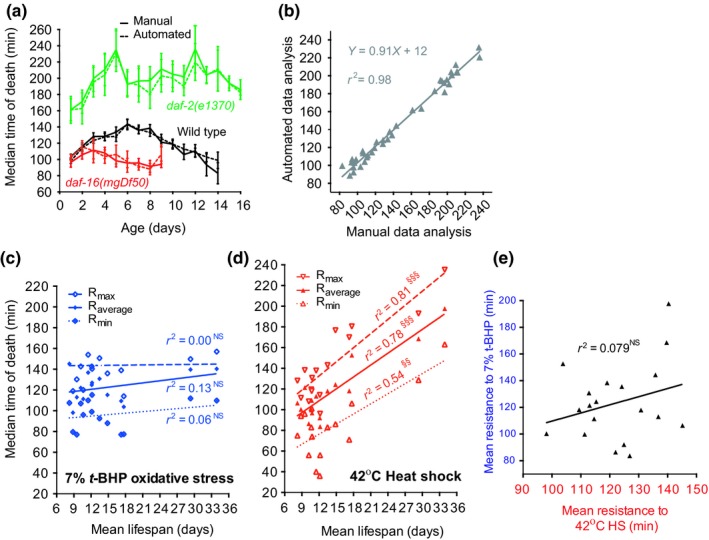
LFASS reveals a strong correlation between severe thermal—but not oxidative—stress resistance and longevity. (a) LFASS automated data analysis package and manual analysis of severe thermal stress resistance yield near‐identical results, and correlate almost perfectly (b). (c) Minimum, mean, and maximum severe oxidative stress resistance does not correlate with mean lifespan, while (d) minimum, mean, and maximum severe thermal stress resistance correlates well with mean lifespan, across the conditions tested. (e) Severe oxidative and thermal stress mean resistance does not correlate. § *p* < 0.05, §§ *p* < 0.01, §§§§ *p* < 0.0001. Minimum, mean, and maximum resistance is calculated over the first week of adulthood

Data generated by LFASS suggest a closer correlation between senescence and susceptibility to thermal stress than to oxidative stress (Figure 2a,b). To explore this further, we re‐examined the relationship between strain differences in lifespan and stress resistance. To facilitate this, we developed a software package to automatically extract median time of death from DF curves, since manual analysis of high‐throughput data generated by LFASS is very time‐consuming (Figure S3, https://github.com/ABA80/LFASS, updates available on request from A.B.). After querying the user for key assay parameters, fluorescence time‐lapse data are automatically sorted, smoothened, fitted, and median times of death logged into a data output table. The <5% unfitted data can be reanalyzed individually with user guidance. Automated analysis yielded near‐identical results to manual analysis (Figure 3a,b) but in ~1/100th of the time, which we took advantage of for further screening (Figure S4).

Regression analysis of severe stress resistance and lifespan data showed a strong positive correlation between longevity and resistance to high thermal stress, but not severe oxidative stress (Figure 3c,d, Table S5). Moreover, there was no correlation between resistance to severe heat and oxidative stresses (Figure 3e). This argues against the importance of oxidative stress as a determinant of lifespan but is consistent with a role in aging of features of cellular function that are sensitive to heat stress, such as protein‐folding homeostasis.

### 2.6 LFASS can be used to score survival in parasitic nematode species

Beyond its utility for *C. elegans* research, we wondered whether LFASS could also be used with parasitic worms, for example, to facilitate anthelmintic drug screens. DF has been documented in several other free‐living nematode species (*C. briggsae*, *Pristionchus pacificus*) (Coburn et al., 2013), but its occurrence in parasitic nematodes has not been investigated. To investigate this, we first tested for blue DF in *Trichuris muris*, *Nippostrongylus brasiliensis*, and *Heligmosomoides polygyrus*, killed with 7% (by volume) *t*‐BHP. DF was not measurable in adult nematodes (data not shown), and we could not collect enough *T. muris* L1 larvae to perform LFASS assays, but DF was detected in *N. brasiliensis* and *H. polygyrus* L3 larvae (comparable in morphology and size to *C. elegans* adults) (Figure S5a,b). In each case, LFASS data showed a DF peak similar to *C. elegans*, but at slightly different wavelengths (Figure S5b) which might reflect differences in tryptophan‐derived metabolite content. However, 42˚C heat shock failed to kill either species (perhaps because it is only 4–5°C above their hosts' body temperature), while *H. polygyrus* showed resistance to *t*‐BHP, possibly due to the protective double cuticle of its L3 stage (Figure S5c). *N. brasiliensis* L3 were readily killed and assayed by LFASS when exposed to 7% *t*‐BHP (Figure S5d). Overall, these results show the potential utility of LFASS for survival assays of larval stages of parasitic nematodes, which could greatly facilitate automated, high‐throughput anthelmintic screens.

## 3 DISCUSSION

In this study, we have used the phenomenon of death fluorescence to develop LFASS, a method for measuring nematode survival under stress (Figure 1). LFASS can be readily conducted using standard fluorometric plate readers to generate high‐throughput data with high reproducibility. To illustrate the application of LFASS, we have used it here a) to test the effects of aging on severe stress resistance; b) to test the effects of mutations that affect lifespan on severe stress resistance; and c) to test the efficacy of LFASS for use with parasitic nematodes of medical and agricultural importance.

LFASS is unbiased, easily implemented, and versatile, requiring no added reagents and is compatible with transgenic, frail, or immobile worms. It does not require a strict sample size so that worm loading could be automated (e.g., using worm sorters and automatic dispensers). It is potentially applicable to modern screening platforms employing transparent materials (e.g., microfluidic chips and multi‐well plates) and should be highly cost‐effective for toxico‐pharmacological studies and genetic screens in the contexts of infection, stress resistance, and aging. Because it can be applied to some mammalian parasitic nematodes species, it could also be used to accelerate anthelmintic drug discovery. A limitation of LFASS is that DF decays over a few hours (Coburn et al., 2013). LFASS is therefore most effective for shorter assays (less than one day). It also means that while assays of less than a couple of hours duration require as few as 16 worms or less per condition, longer assays (e.g., bacterial infection) require larger numbers of worms (>100 worms per well). This is because the interval between consecutive death events needs to be short enough for individual worm fluorescence signals to add up and produce a clear DF peak at population level.

Yet, because of the ease with which LFASS can gather data at short time intervals, we were able to accurately measure the effects of aging on resistance to high levels of stress that result in a short survival time. Strikingly, this revealed early adulthood increases in severe stress resistance associated with age in all cases, which in the case of oxidative stress increased for much of adulthood (Figure 2).

These results contrast sharply with those obtained using more moderate stress paradigms, where resistance typically declined with age from the onset of adulthood (Darr & Fridovich, 1995; Labbadia & Morimoto, 2015; Youngman et al., 2011). One possible reason for differences in responses to severe and moderate stressors is that the rapidity with which severe stress impairs biological function precludes the development of a timely adaptive response (e.g., increased levels of antioxidant enzymes and heat‐shock protein levels, or increased protein turnover). By this view, severe stress resistance levels may more closely correspond to preset/intrinsic (as opposed to induced/adaptive) stress responses (Lithgow, White, Hinerfeld, & Johnson, 1994). A further possibility is that severe stress resistance reflects resistance to the organismal death cascade (Galimov et al., 2018).

Our findings suggest that the mechanisms of intrinsic resistance differ between stress modalities, and in how they change with age. The different timings of peak resistance to severe infection (day 4), oxidative (day 12), and thermal (day 6) stress make pinpointing peak health in adulthood challenging, particularly as severe aging pathologies develop while severe thermal and oxidative stress resistance is still increasing (Ezcurra et al., 2018) (Figure S6).

Interestingly, LFASS profiles imply that autophagy is required for much of the age‐dependent increase in severe stress resistance, apart from oxidative stress sensitivity in very early adulthood, where reducing autophagy increases resistance. It is surprising that autophagy activity initially sensitizes young adults to (up to day 4), but later protects middle‐aged worms against (days 5–12) severe oxidative stress. Autophagy also promotes the conversion of intestinal biomass into yolk, resulting in gut atrophy (Ezcurra et al., 2018); one possibility here is that by promoting intestinal senescence, autophagy increases susceptibility to oxidative stress. The results imply that first detrimental and then beneficial effects of autophagy predominate. This underscores the double‐edged role played by autophagy in *C. elegans* aging (Benedetto & Gems, 2019; Ezcurra et al., 2018; Shintani & Klionsky, 2004).

Interestingly, comparison of strain differences in average resistance (over the first week of adulthood) to severe stress and lifespan showed that resistance to high temperature but not severe oxidative stress is strongly positively correlated with longevity. This is consistent with many recent studies that have argued against the importance of oxidative stress as a major cause of aging in *C.* *elegans* (Gems & Doonan, 2009; Van Raamsdonk & Hekimi, 2010), and for that of protein‐folding homeostasis (Garigan et al., 2002; Hsu, Murphy, & Kenyon, 2003; Labbadia & Morimoto, 2014; Morley & Morimoto, 2004). It also supports the idea that thermal stress resistance is a good predictor of longevity (Munoz & Riddle, 2003).

In conclusion, our results paint a complex picture of the nuanced relationship between stress sensitivity and advancing age, where animals of a given age can exhibit increased resistance to one stress modality and hypersensitivity to another (Figure S6). To an extent, they challenge the assumption that aging is a process of loss of homeostasis from early adulthood and that one can understand *C. elegans* lifespan in terms of clear phases of progressive decline (Bansal, Zhu, Yen, & Tissenbaum, 2015; Huang, Xiong, & Kornfeld, 2004). Understanding the underlying mechanisms could prove useful in human contexts such as extreme sport practice (marathon running, high‐altitude climbing, deep diving), acute poisoning, and exposure to heat waves. These findings illustrate the utility of LFASS for exploring new facets of nematode biology, here yielding fresh insights into the biology of aging.

## 4 EXPERIMENTAL PROCEDURES

### 4.1 *C. elegans* culture conditions

*Caenorhabditis elegans* strains were maintained at 15˚C following standard culture conditions (Brenner, 1974), on NGM agar plates seeded with *E. coli* OP50. Aging worm cohorts were prepared as follows. Young adult hermaphrodites (30 per plate) were allowed to lay eggs for 24 hr. Several days later, L4 animals were collected and transferred to NGM/OP50 plates containing 15 µM fluorodeoxyuridine (FUDR) (Sigma‐Aldrich #F0503) to block egg production, or to NGM with 15 µM FUDR and 25 µg/ml carbenicillin (Sigma‐Aldrich #C3416), and seeded with HT115 RNAi‐producing bacteria, as described (Kamath & Ahringer, 2003), and in each case maintained at 25˚C. L4 larvae were collected in this way daily for 2–3 weeks, and then, adult hermaphrodites of each age were picked into multi‐well plates and subjected to LFASS all on the same day. For more details, see Supporting Information.

### 4.2 Parasitic nematode handling

*H. polygyrus* was maintained by passaging through female C57BL/6J mice as described (Filbey et al., 2018; Hayes et al., 2017). *T. muris* was maintained in genetically susceptible mice as described (Hayes et al., 2017). *N. brasiliensis *was maintained by passaging through male Sprague‐Dawley rats as described (Lawrence, Gray, Osborne, & Maizels, 1996). For full details, see Supporting Information.

### 4.3 Time‐lapse microscopy experiments

100–120 1‐day‐old adult hermaphrodites were mounted in M9 on 2% agarose pads between slide and coverslip without anesthetic unless otherwise stated. Imaging was performed through a DAPI filter set (Chroma Technology Corp, USA) using a 2.5× objective on a Leica DMRXA2 microscope (Leica Biosystems Nussloch GmbH, Germany). Successive bright‐field and DAPI images were acquired every 30 s using the Volocity 6.3 software (Perkin Elmer, USA). For further details, see Supporting Information.

### 4.4 Time‐lapse microscopy analysis

We used the Volocity 6.3 Quantitation module to generate graphic representations (kymographs) of single worm traces from the 2.5× time‐lapse imaging series. The time of death for each worm was deduced from the time of the intestinal blue fluorescence burst. Individual times of death during a single time‐lapse were fitted into bins and count distributions plotted and fitted with a Gaussian curve using GraphPad Prism 6.0 software (GraphPad Software Inc., USA). Overall fluorescence for each time point was measured using the ImageJ‐based open‐source package Fiji (http://fiji.sc/Fiji), plotted, and analyzed using GraphPad Prism 6.0.

### 4.5 Plate‐reader assays

For oxidative stress and heat‐shock assays, we picked 16 worms into 60 µl M9 per well for 384‐well plates, and 50 worms in 150 µl M9 for 96‐well plates, together with a pellet of *E. coli* OP50 bacteria to prevent starvation. For infection assays, *Enterococcus faecalis* GH10 bacteria were streaked onto brain–heart infusion kanamycin (BHIK) agar plates and used within a week, as described (Garsin et al., 2001). Liquid (BHI) *E. faecalis* cultures were grown for 3–5 hr at 37°C to saturation on the day. We then picked 100 worms per well into 50 µl M9 + 30 µl OP50 medium (for 384‐well plates), and supplemented with 10 µl freshly saturated *E. faecalis* solution cooled to room temperature. A Tecan Infinite 200 plate reader (Tecan Group Ltd., Switzerland) was prewarmed at 25°C to match the temperature at which aged cohorts were raised and *E. faecalis* infections assays performed. Blue fluorescence (excitation: 360 nm/emission: 435 nm) was recorded for each well every 2 min for 8 hr or every 5 min for 4 days for stress and infection assays, respectively.

### 4.6 Death fluorescence (DF) curve manual analysis

Fluorescence time‐lapse recording data for each well were normalized. The maximum was chosen where a significant peak of fluorescence was observed. After normalization, the time of half‐maximum fluorescence was determined. For full details, see Supporting Information.

### 4.7 DF curve automated analysis

MATLAB 2014b and 2015a versions were used to write and execute the LFASS software package. Figure S3 describes the approach. For details, see Supporting Information.

### 4.8 Lifespan assays

With the exception of RNAi experiments, all worm cohorts used in reported stress or lifespan assays were hermaphrodites maintained at 15°C on OP50‐seeded NGM plates and switched at the L4 stage to OP50 plates supplemented with 15 µM FUDR, and subsequently maintained at 25°C.

### 4.9 Statistics

For lifespan statistics, we used the JMP 12.01 Pro software package from SAS (USA). Lifespans were compared using the nonparametric log‐rank test. Unless otherwise stated, all other statistics were performed using Prism 6.0 from GraphPad Software Inc. (USA). Stress resistance differences with age and across genotypes were assessed by two‐way ANOVA with post hoc Dunnett's test. *p* values reported in supplementary tables are adjusted for multiple comparisons.

## CONFLICT OF INTEREST

None declared.

## AUTHOR CONTRIBUTIONS

AB conceived the study, and designed and supervised experiments, with input from FC and DG. CA, AB, TB, BC, FC, KC, HD, JM, and JMAT performed experiments. AB conducted analysis and interpretation of data, with input from FC and DG. AB and DG wrote the manuscript.
