# Supplementary material for: New label‐free automated survival assays reveal unexpected stress resistance patterns during C. elegans aging
Source: Aging Cell. 2019 Jul 16;18(5):e12998. doi: 10.1111/acel.12998 (PMC6718543; doi:10.1111/acel.12998)
Supplement: Supplementary file 1 [file ACEL-18-e12998-s001.pdf]

Supporting Information

A. Benedetto *et al*. (2019)
New label-free automated survival assays reveal unexpected stress resistance patterns during *C. elegans* ageing

Supplemental Experimental Procedures

C. elegans strains and handling

The following strains were used in this study: N2 (wild type); DA597, *phm-2(ad597) I*; DA2123, *adIs2122 [lgg-1p::GFP::lgg-1 + rol-6(su1006)]*; DR1567, *daf-2(m577) III*; DR1572, *daf-2(e1368) III*; EFS7, *daf-16(mgDf50) I*; *daf-2(e1370) III*; EU1, *skn-1(zu67) IV/nT1 [unc-?(n754) let-?] (IV;V)*; GA60, *eat-2(ad1116) II*; GA82, *daf-2(e1370) III*; GA91, *daf-16(mgDf50) I*; *daf-2(m577) III*; GA1001, *aak-2(ok524) X*; GR1307, *daf-16(mgDf50) I*; GS776, *unc-32(e189) lin-12(n676n930) III; unc-42(e270) sel-11(ar39) V*; GS807, *unc-32(e189) lin-12(n676n930) III; unc-42(e270) sel-11(ar39) V*; HZ1683, *him-5(e1490) V; atg-2(bp576) X*; HZ1684, *atg-3(bp412) IV; him-5(e1490) V*; HZ1685, *atg-4.1(bp501) I*; HZ1686, *bnIs1 I; atg-7(bp411) IV; him-5(e1490) V*; HZ1687, *atg-9(bp564) him-5(e1490) V*; HZ1688, *him-5(e1490) V; atg-2(bp576) X*; LD1004, *Ex001[SKN -1B/C::GFP]*; PS3551, *hsf-1(sy441) I*; RE666, *ire-1(v33) II*; RB545, *pek-1(ok275) X*; RB938, *vha-12(ok821) X*; RB1021, *crt-1(ok948) V*; SJ17, *xbp-1(zc12) III; zcIs4 V*; SJ30, *ire-1(zc14) II; zcIs4 V*; SJ4005, *zcIs4[hsp-4::GFP] V*; SJ4100, *zcIs13[hsp-6::GFP]*; ST6, *eat-20(nc4) X*; VC475, *hsp-16.2(gk249) V*; VC893, *atg-18(gk378) V*; ZG31, *hif-1(ia4) V*. GA60, GA82, GA91, GA1001 were generated in the Gems lab. LD1004 was kindly provided by Keith Blackwell, and EFS7 by Eugene Schuster. All other strains used in this study were provided by the *Caenorhabditis* Genetics Center, University of Minnesota). *C. elegans* strains were maintained at 15˚C following standard culture conditions (Brenner, 1974), on NGM 60 mm-diameter agar plates seeded with *E. coli* OP50. Aging worm cohorts were prepared as follows. Young adult hermaphrodites (30 per plate) were allowed to lay eggs for 24 hr. Several days later L4 animals were collected and transferred to NGM/OP50 plates containing 15 µM fluorodeoxyuridine (FUDR) (Sigma-Aldrich ♯F0503) to block egg production; or to NGM with 15µM FUDR and 25µg/mL carbenicillin (Sigma-Aldrich ♯ C3416), and seeded with HT115 RNAi-producing bacteria, as described (Kamath & Ahringer, 2003), and in each case maintained at 25˚C. L4 larvae were collected in this way daily for 2-3 weeks, and then adult hermaphrodites of each age were picked into multi-well plates and subjected to LFASS all on the same day.

Parasitic nematode handling

H. polygyrus was maintained by passaging through female C57BL/6J mice. Mice were dosed with 200 infective L3 larvae by oral gavage. Faecal pellets were collected 3-4 weeks post infection and cultured as described (Filbey et al., 2018; Hayes et al., 2017). Infective third (L3) stage larvae were collected and stored in tap water at 4˚C. Larvae remain infective for up to 6 months, after which infectivity declines.

T. muris was maintained in genetically susceptible mice as described(Hayes et al., 2017). Adult worms were collected from the caecum of culled mice at 42 days post-infection

and cultured for 4 hours at 37˚C in RPMI 1640 containing 2% FCS, 500 IU/ml penicillin, 500µg/ml streptomycin and 2mM L-glutamine.

N. brasiliensis was maintained by passaging through male Sprague-Dawley rats. Fecal pellets were collected on days 5-7 post-infection and cultured as described (Lawrence, Gray, Osborne, & Maizels, 1996). Third stage (L3) larvae were collected using a Baermann apparatus and washed 10x in PBS (1x) and kept at 15˚C in PBS (1x) with Nystatin 120U/mL.

Time-lapse microscopy experiments

100-120 1 day old adult hermaphrodites were mounted in M9 on 2% agarose pads between slide and coverslip without anaesthetic unless otherwise stated. Imaging was performed through a DAPI filter set (Chroma technology Corp, USA) using a 2.5x objective on a Leica DMRXA2 microscope (Leica Biosystems Nussloch GmbH, Germany). Successive brightfield and DAPI images were acquired every 30 s using the Volocity 6.3 software (Perkin Elmer, USA). For rapid killing experiments (Figure S1a), a 70% solution of *tert*-butyl-hydroperoxide (Luperox TBH70X, Sigma-Aldrich #458139, Switzerland) was added volume to volume to the mounting M9 medium prior to coverslip apposition. The imaging protocol was started exactly 1 min later. For heat-killing experiments (Figure S1b), a PE120 heating/cooling platform (Linkam Scientific Instruments Ltd, UK) was attached to the microscope stage. The heating protocol (ramping up to 42˚C at 1.2˚C per min) was initiated simultaneously with the time-lapse imaging.

Time-lapse microscopy analysis

We used the Volocity 6.3 Quantitation module to generate graphic representations (kymographs) of single worm traces from the 2.5x time-lapse imaging series. The time of death for each worm was deduced from the time of the intestinal blue fluorescence burst. Individual times of death during a single time-lapse were fitted into bins and count distributions plotted and fitted with a Gaussian curve using GraphPad Prism 6.0 software (GraphPad Software Inc., USA). Overall fluorescence for each time point was measured using the ImageJ-based open-source package Fiji (http://fiji.sc/Fiji), plotted and analyzed using GraphPad Prism 6.0.

Plate-reader assays

For oxidative stress and heat shock assays, we picked 16 worms into 60 µL M9 per well for 384-well plates, and 50 worms in 150 µL M9 for 96-well plates, together with a pellet of *E. coli* OP50 bacteria to prevent starvation. For infection assays, *Enterococcus faecalis* GH10 bacteria were streaked onto Brain Heart Infusion Kanamycin (BHIK) agar plates and used within a week, as described (Garsin et al., 2001). Liquid (BHI) *E. faecalis* cultures were grown for 3-5 h at 37˚C to saturation on the day. We then picked 100 worms per well into 50 µL M9 + 30 µL OP50 medium (for 384-well plates), and supplemented with 10 µL freshly saturated *E. faecalis* solution cooled to room temperature. A Tecan Infinite 200 plate-reader (Tecan Group Ltd., Switzerland) was pre-warmed at 25˚C to match the temperature at which aged cohorts were raised and *E. faecalis* infections assays performed. Blue fluorescence (excitation: 360 nm / emission: 435 nm) was recorded for each well every 2 min for 8 h or every 5 min for 4 days for stress and infection assays, respectively.

Death fluorescence (DF) curve manual analysis

Fluorescence time-lapse recording data for each well were normalised. The maximum was chosen where a significant peak of fluorescence was observed. Fluorescence values for the first 15-20 time points were often inaccurate, yielding local maxima and minima, due to worm thrashing in the wells, and were therefore omitted for the determination of the fluorescence minimum and maximum. After normalization, the time of half-maximum fluorescence was determined.

DF curve automated analysis

Matlab 2014b and 2015a versions were used to write and execute the LFASS software package. Figure S3 describes the approach. Detailed documentation (description of functions and variables) is provided together with the package (LFASS.zip) within the Readme_LFASS.txt file. Briefly, the program proceeds as follow.

(1) Matlab separates text and number matrices so that tags and values are stored in separate matrices. In .xlsx plate-reader files each row represents the fluorescence of a single well over time. The last column is the well identity/tag (attributes of the sample: age, genotype, drug treatment, bacterial type).

(2) For the fit to perform optimally and return median time of death in minutes, several assay parameters have to be informed by the user. The time interval between two measurements of the same well allows for the results to be expressed in minutes. The noise threshold allows for discarding empty wells, and wells in which no fluorescence peak can be detected. The noise fluorescence threshold should be chosen above the fluorescence values measured in empty wells, and below the peak blue fluorescence value sample-containing wells. This threshold also depends on the number of worms per well, and all data treated in the same bulk analysis should have roughly the same number of worms per well. Max and min have to be identified for the normalisation of the data. Early fluorescence values can greatly fluctuate due to worm thrashing/swimming (in the absence of anaesthetic) associated with high or low fluorescence values that can exceed the relevant maximum and minimum. For this reason, the user must indicate in which time intervals min and max are to be expected. These intervals usually exclude the first 10-20 time points and have to include all the times of minimum or maximum fluorescence of all the data sets included in one analysis. Once the data are normalised, the sigmoid fit needs to be constrained within initial and final plateaux that match the min and the max values. Because of inaccuracies in measurements, to choose the best plateaux, they need to be fitted over several time points. To achieve this, a tolerance threshold is given for the min/max (i.e., 0.5/0.95). The fit function will then take into account time points around the min/max that are found within these tolerance thresholds (between 0 and 0.5, and between 0.95 and 1, respectively) and stop looking for additional points beyond. This will effectively define a fit region that encompasses the death-associated blue fluorescence burst, ignoring all other parts of the curve (see green dotted lines in Figure S3 (3b) and (b) lower panels).

(3) To speed up computing, the program first excludes rows that do not need to be fitted such as parameter rows (date, time points, temperature with time, etc.) by keeping only rows with at least 6 consecutives numbers (non-data rows contain letters). It then uses the noise threshold defined earlier to exclude data rows that do not have values above this threshold, which eliminates most of the empty wells (3a). Inaccuracies in measurements are associated with noise spikes that can complicate fitting. To limit their influence, the data are smoothened twice using the "smooth" function (4 other smoothing options were compared, and this performed best).

Min and max are found, data are normalized, and a fit interval is found. In the region to fit, curves are typically sigmoidal in shape. Because we are only interested in extracting the time of half-maximum (corresponding to the median time of death, Figure S1), the critical region to fit is around this time point, and the sigmoid fit performs optimally.
(4) As the analysis progresses, a 4-column wide .txt result table is filled. Column 1 contains all the tags, column 2 reports the half-maximum time inferred from unfitted normalised raw data, column 3 reports half-maximum times obtained with the bulk fitting analysis, and column 4 reports the updated values obtained from bulk fitting and user-guided analyses. Dataset names and parameters (temperature, duration of assay) are filled in the first rows of column 1. "0" fill empty cells from non-data rows. "1" fill cells in data rows that did not pass the user-defined noise threshold. "NaN" fills cells in data rows that could not be fitted.
(5) When the fit does not converge for a given row, the user can re-analyse it giving attribute values that differ from the bulk analysis and that are better suited for this specific row. Typically, this allows for recovery of the 5% exploitable low-quality data that are excluded by the bulk analysis. The result table is updated after each re-analysis until the user stops.
(6) Post-processing is performed in Microsoft Excel for data sorting and basic row/columns operations. Then statistical analysis and graphical representations are processed in GraphPad Prism.

Lifespan assays

With the exception of RNAi experiments, all worm cohorts used in reported stress or lifespan assays were hermaphrodites maintained at 15˚C on OP50-seeded NGM plates and switched at the L4 stage to OP50 plates supplemented with 15μM FUDR, and subsequently maintained at 25˚C.

Statistics

For lifespan statistics we used the JMP 12.01 Pro software package from SAS (USA). Lifespans were compared using the non-parametric log rank test. Unless otherwise stated, all other statistics were performed using Prism 6.0 from GraphPad Software Inc. (USA). Stress resistance differences with age and across genotypes were assessed by two-way ANOVA with a post-hoc Dunnett's test. *p* values reported in supplementary tables are adjusted for multiple comparisons.

REFERENCES

Brenner, S. (1974). The genetics of Caenorhabditis elegans. *Genetics, 77*, 71-94.

Cabreiro, F., Au, C., Leung, K. Y., Vergara-Irigaray, N., Cocheme, H. M., Noori, T., . . . Gems, D. (2013). Metformin retards aging in C. elegans by altering microbial folate and methionine metabolism. *Cell, 153*, 228-239.

Ezcurra, M., Benedetto, A., Sornda, T., Gilliat, A. F., Au, C., Zhang, Q., . . . Gems, D. (2018). C. elegans eats its own intestine to make yolk leading to multiple senescent pathologies. *Curr. Biol. 28*, 2544-2556.

Filbey, K. J., Camberis, M., Chandler, J., Turner, R., Kettle, A. J., Eichenberger, R. M., . . . Le Gros, G. (2018). Intestinal helminth infection promotes IL-5- and CD4(+) T cell-dependent immunity in the lung against migrating parasites. *Mucosal Immunol. 12*, 352-362.

Garsin, D. A., Sifri, C. D., Mylonakis, E., Qin, X., Singh, K. V., Murray, B. E., . . . Ausubel, F. M. (2001). A simple model host for identifying Gram-positive virulence factors. *Proc Natl Acad Sci U S A, 98*, 10892-10897.
Hayes, K. S., Cliffe, L. J., Bancroft, A. J., Forman, S. P., Thompson, S., Booth, C., & Grencis, R. K. (2017). Chronic Trichuris muris infection causes neoplastic change in the intestine and exacerbates tumour formation in APC min/+ mice. *PLoS Negl Trop Dis, 11*, e0005708.
Kamath, R. S., & Ahringer, J. (2003). Genome-wide RNAi screening in Caenorhabditis elegans. *Methods, 30*, 313-321.
Lawrence, R. A., Gray, C. A., Osborne, J., & Maizels, R. M. (1996). Nippostrongylus brasiliensis: cytokine responses and nematode expulsion in normal and IL-4-deficient mice. *Exp Parasitol, 84*, 65-73.
Riesen, M., Feyst, I., Rattanavirotkul, N., Ezcurra, M., Tullet, J. M., Papatheodorou, I., . . . Gems, D. (2014). MDL-1, a growth- and tumor-suppressor, slows aging and prevents germline hyperplasia and hypertrophy in C. elegans. *Aging, 6*, 98-117.
Tullet, J. M., Araiz, C., Sanders, M. J., Au, C., Benedetto, A., Papatheodorou, I., . . . Gems, D. (2014). DAF-16/FoxO directly regulates an atypical AMP-activated protein kinase gamma isoform to mediate the effects of insulin/IGF-1 signaling on aging in Caenorhabditis elegans. *PLoS Genet, 10*, e1004109.

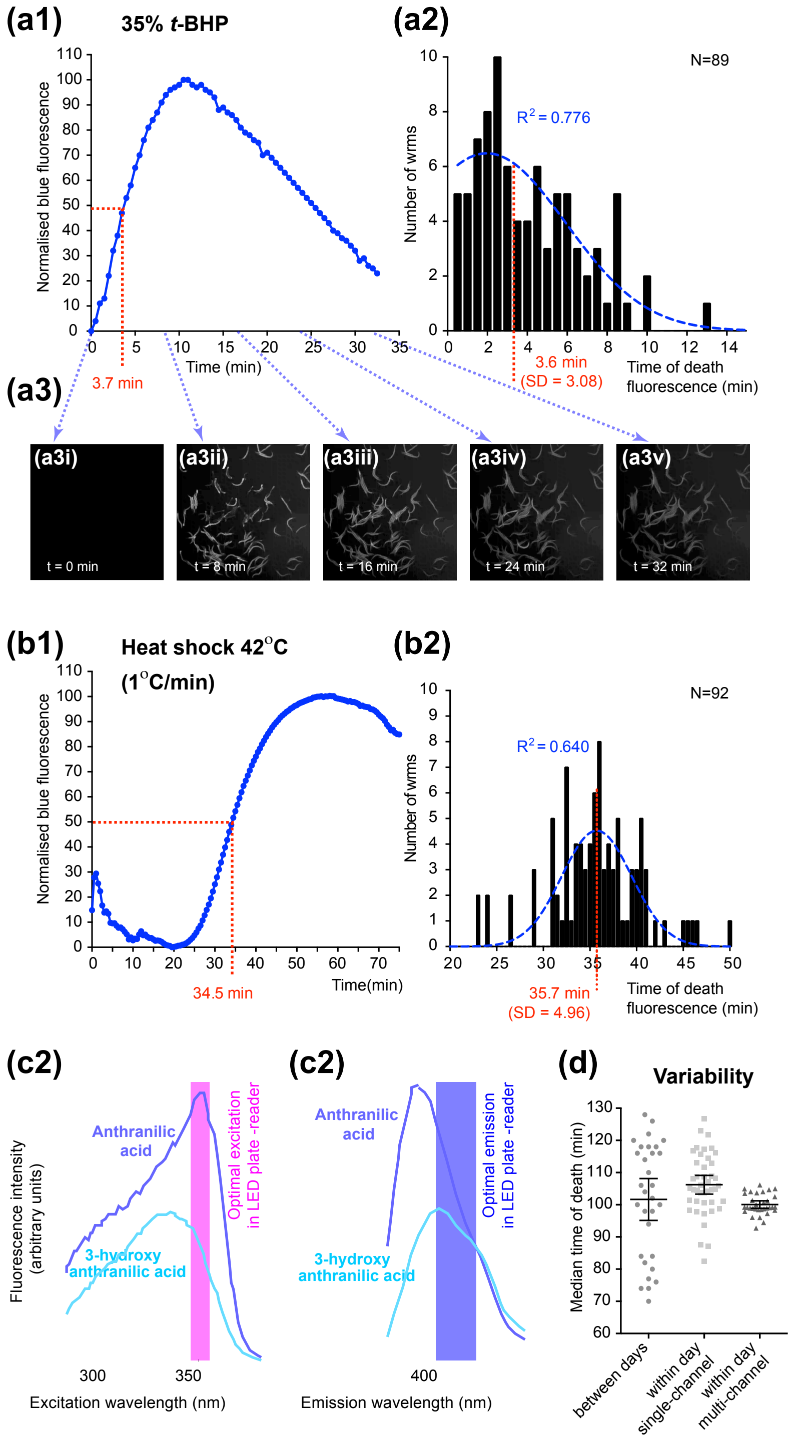

Figure S1 | Using population death fluorescence to determine median time of death. Time-lapse microscopy (exemplified by panels a3i-a3v) of worm population submitted to fast (a) and slower (b) killing assays reveal that the time of half maximum death fluorescence (a1, b1, red dotted line) corresponds to the median time of death (a2, b2, red dotted line). Optimal excitation (c1) and emission (c2) windows for anthranilate-dependent death fluorescence in 384 well plates read by a monochromator-based plate-reader, matched with excitation and emission spectra for two commercially available anthranilate compounds. (d) Sources of variability when measuring median time of death by LFASS. Interday variability is much greater than intraday variability between age-matched samples, while sequential single channel pipetting leads to more variable measurements than multichannel pipetting.

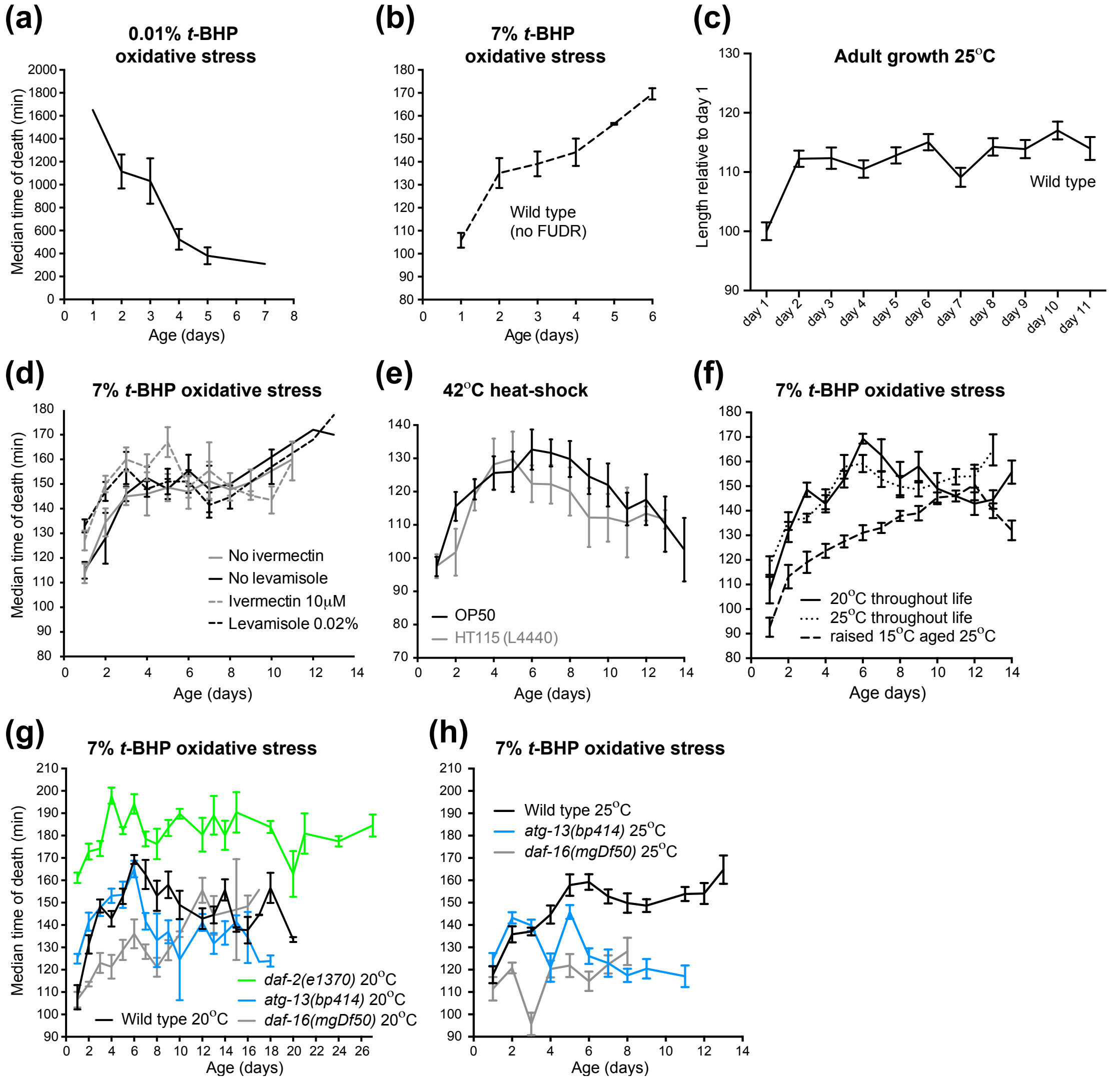

Figure S2 | Additional control experiments. (a) Adult resistance to milder oxidative stress (0.01% instead of 7% *t*-BHP) decreases with age during the first week of adulthood. (b) The age-associated increase in severe oxidative stress resistance occurs also in absence of FUDR. (c) Worms aged at 25˚C do not grow significantly beyond day 2 (the increase in severe stress resistance beyond day 2 is not a direct consequence of growth or size). (d) Inhibition of pharyngeal pumping by ivermectin or levamisole does not suppress the age increase in severe oxidative stress resistance. (e) The pattern of severe heat resistance with age is the same on the two common yet distinct *E. coli* diets (OP50 and HT115). (f) The increase of severe oxidative stress resistance with age is observed across various temperature conditions (it does not simply result from an hormetic process following a shift from a lower to a higher temperature). The relative effects of *daf-2*, *daf-16* and *atg-13* inhibitions on severe oxidative stress resistance patterns with age are conserved when animals are grown and aged at 20˚C (g) or 25˚C (h). *daf-2(e1370)* could not be assessed throughout life at 25˚C as it enters into the dauer stage at this temperature (which is why animals were kept at 15˚C and transferred at 25˚C at L4 stage for most aging experiments in this study).

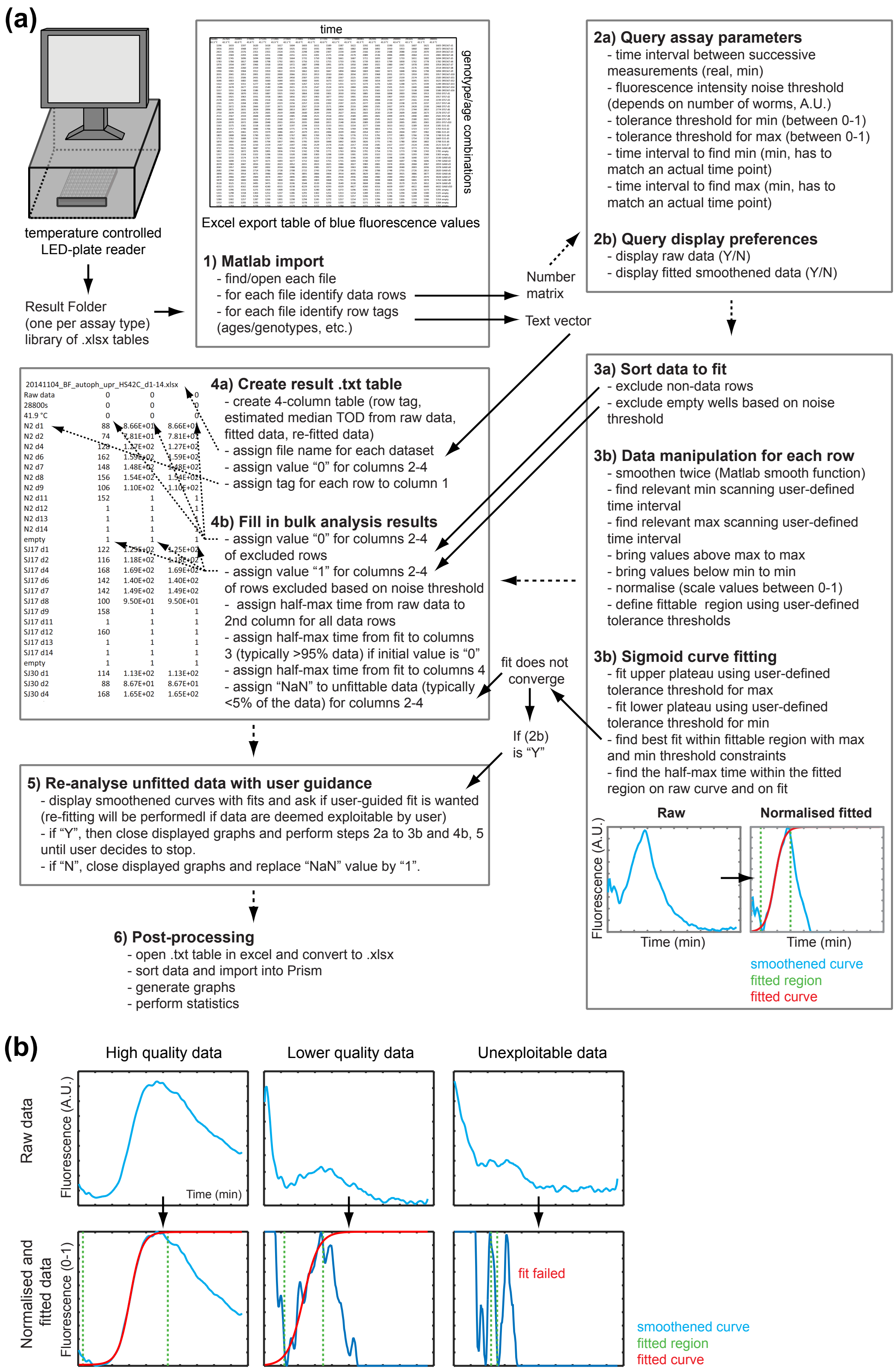

Figure S3 | Diagram of the automated curve analysis package. (a) Sequence of operations performed to automatically extract median time of death from raw time-lapse fluorescence data acquired by the monochromator-based plate-reader. (b) Examples of typical fluorescence curves and how the automated bulk analysis fits them. For bulk analysis, the same assays parameters are given for all data. The fitted region (green) adapts to the data, allowing for a good fit (red) to be found even on lower quality data (middle panels). Note that data deemed non-exploitable by visual inspection are not fitted (right panels) preventing erroneous data from filling the result table.

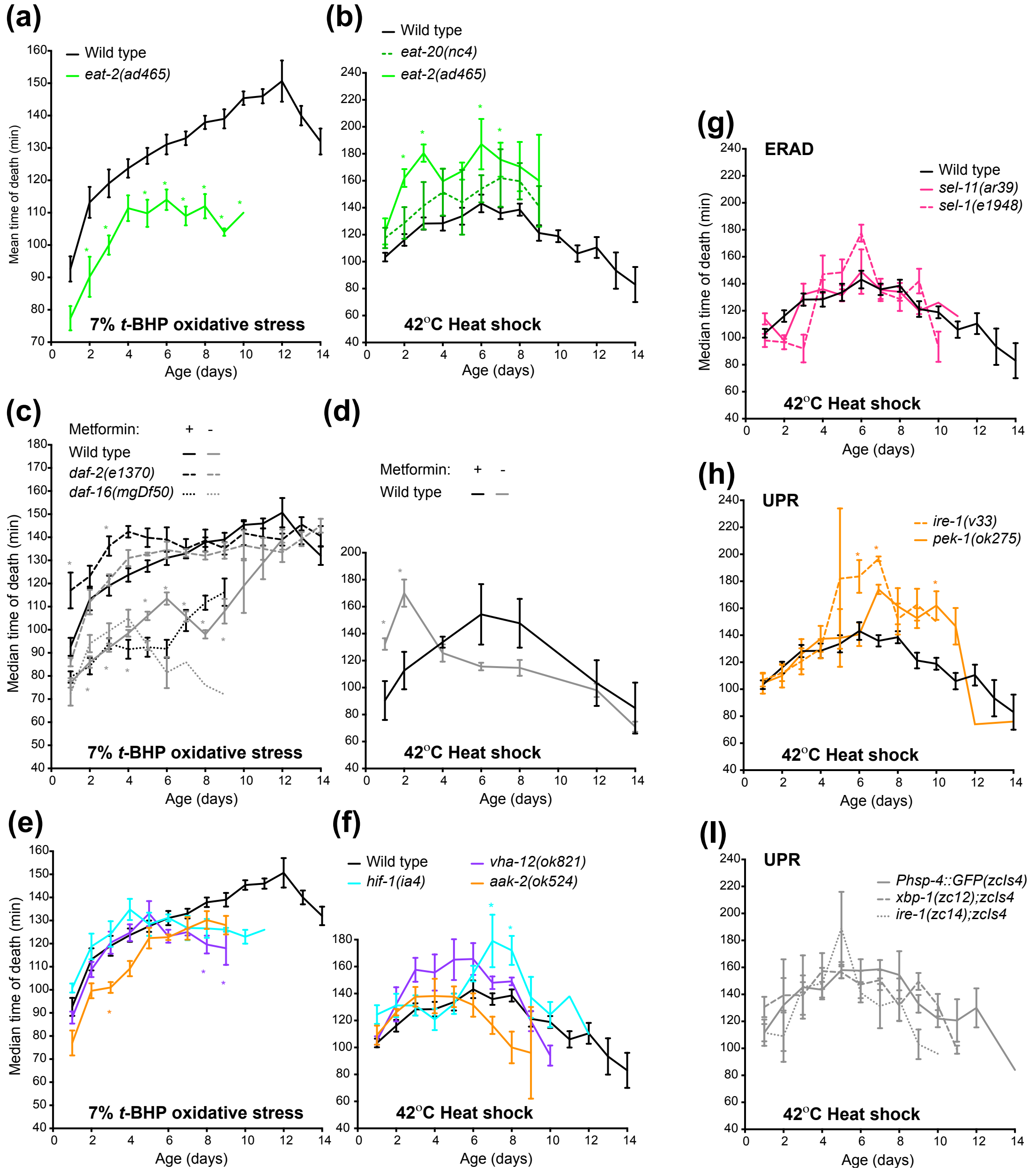

Figure S4 | Impact of various stress associated gene mutations and drug treatments on patterns of severe stress resistance with age. (a,b) Slower pumping rate and associated genetically-induced dietary restriction (DR) do not correlate with severe oxidative-stress resistance dynamics (a) but correlates with heat-shock resistance dynamics (b): *eat-20(nc4)* is a mild pumping-defective mutant, *eat-2(ad465)* is a strong pumping/genetic DR mutant, *daf-2(e1370)* pumps more slowly than *daf-2(m577)* and so combines a genetic DR phenotype with reduced IIS (see Figure 1). (c,d) The lifespan extending drug metformin sensitizes *daf-2* and wild type worms to severe oxidative stress but confers increased heat-shock resistance to young wild-type adults. (e,f) AMP-activated protein kinase alpha subunit (*aak-2*), vacuolar-H^+-ATPase subunit 12 (*vha-12*) and hypoxia-induced factor 1 (*hif-1*) differentially affect age increases in severe oxidative-stress and heat-shock resistance. (g-i) The endoplasmic reticulum-associated protein degradation (ERAD) and unfolded protein response (UPR) pathways are dispensable for the age increase in severe thermal stress resistance. Error bars, SEM. Comparison with age-matched wild-type: * p<0.05 down to p<0001.

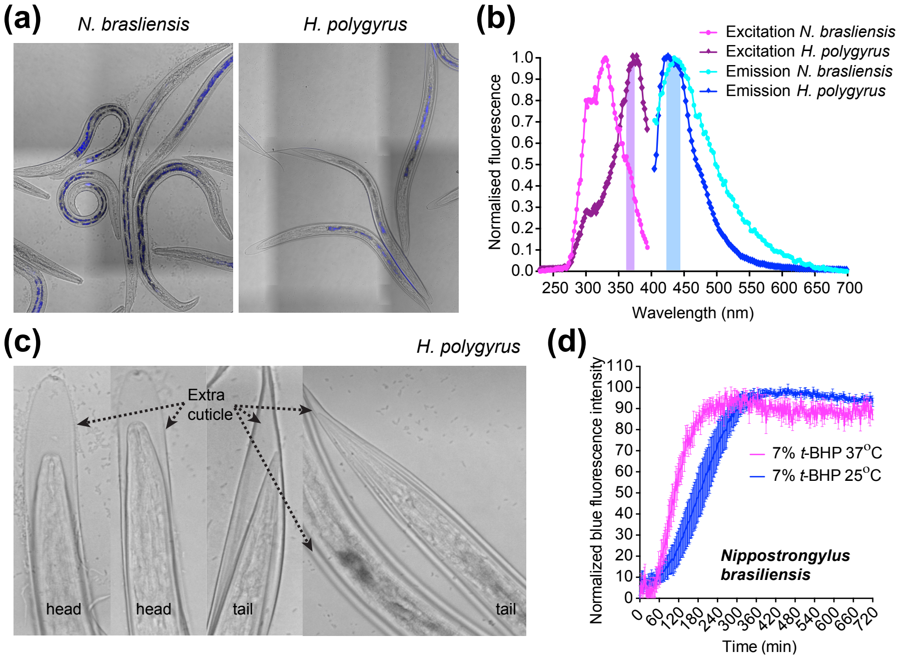

Figure S5 | Applicability of LFASS to mammalian parasitic nematodes *H. polygyrus* and *N. brasiliensis*. (a) Dying L3 larvae of *H. polygyrus* and *N. brasiliensis* display intestinal blue fluorescence. (b) Death fluorescence excitation/emission spectra for *H. polygyrus* and *N. brasiliensis* at L3 stage differ. Worms were killed by freeze-thaw here. The shaded bandwidths correspond to the excitation/emission bands used for LFASS with *C. elegans*. (c) *H. polygyrus* at L3 stage is encased in a double cuticle (arrow heads). (d) LFASS is applicable to *N. brasiliensis* L3. 7% *t*-BHP exposure kills *N. brasiliensis* L3 quicker at 37˚C than at 25˚C.

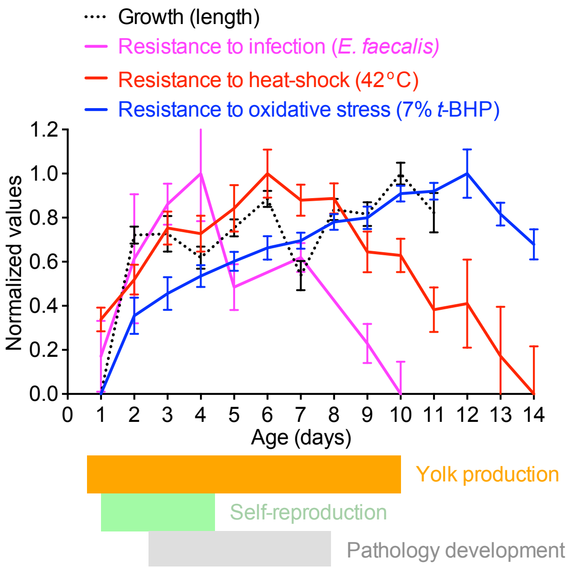

Figure S6 | Normalized juxtaposed dynamics of severe stress resistance during hermaphrodite adulthood at 25˚C. The period of adult growth, self-reproduction, yolk production and senescent pathology development (after which pathologies have reached maximum severity) are indicated below to contextualise severe stress resistance dynamics. Data from this paper and a previous study (Ezcurra et al., 2018).

(a)

Adjusted P values for Dunnett multiple comparison with wild type (2-way ANOVA)	number of independent experiments	day 1	day 2	day 3	day 4	day 5	day 6	day 7	day 8	day 9	day 10	day 11	day 12	day 13	day 14
daf-16(mgDf50)	5	-	-	-	-	0.0476	0.001	0.0102	0.0084	-	NA	NA	NA	NA	NA
daf-16(mgDf50);daf-2(e1370)	5	-	-	0.0068	-	-	-	-	0.0212	-	-	NA	NA	NA	NA
daf-16(mgDf50);daf-2(m577)	5	-	-	-	-	-	-	-	-	-	NA	NA	NA	NA	NA
daf-2(e1370)	9	< 0.0001	< 0.0001	< 0.0001	< 0.0001	< 0.0001	< 0.0001	< 0.0001	< 0.0001	< 0.0001	< 0.0001	< 0.0001	< 0.0001	< 0.0001	< 0.0001
daf-2(m577)	5	-	0.0011	< 0.0001	< 0.0001	0.0053	0.0016	-	-	0.0019	0.0026	< 0.0001	0.0001	0.0006	0.0045
daf-2(e1368)	4	-	-	-	-	-	-	-	-	0.0007	0.0419	0.0002	NA	NA	NA
eat-2(ad465)	5	-	0.0009	0.0008	-	-	0.0031	0.0122	-	-	-	-	NA	NA	NA
eat-20(nc4)	5	-		-	-	-	-	-	-	-	-	-	NA	NA	NA
phm-2(ad597)	5	-	0.0206	-	-	-	-	-	-	-	-	-	NA	NA	NA
skn-1(zu67)	5	-	-	-	-	-	-	-	-	-	-	NA	NA	NA	NA
hsf-1(sy441)	4	-	-	-	-	-	0.0107	-	-	-	-	-	NA	NA	NA
hsp-16.2(gk249)	4	-	-	-	0.0429	-	0.0309	-	-	-	-	0.0288	NA	NA	NA
atg-2(bp576)	14	-	-	-	-	0.0097	0.0086	0.0002	0.0008	-	0.0439	-	-	NA	NA
atg-3(bp412)	9	-	0.01	0.0001	0.0029	-	0.0002	-	-	-	-	-	-	NA	NA
atg-4.1(bp501)	14	-	-	0.0404	-	-	-	-	-	-	-	-	-	NA	NA
atg-7(bp411)	9	-	0.0302	0.0002	0.0224	0.0322	0.0004	-	0.0004	-	-	NA	NA	NA	NA
atg-9(bp564)	9	-	-	-	-	-	0.001	-	-	-	-	-	-	NA	NA
atg-13(bp414)	14	-	0.0016	< 0.0001	< 0.0001	< 0.0001	< 0.0001	< 0.0001	< 0.0001	0.0035	0.0001	-	-	NA	NA
atg-18(gk378)	9	-	-	0.007	-	0.0132	< 0.0001	0.0093	< 0.0001	0.0319	0.0006	-	-	NA	NA
hif-1(ia4)	5	-	-	-	-	-	-	0.0136	0.0376	-	-	-	-	NA	NA
aak-2(ok584)	5	-	-	-	-	-	-	-	-	-	NA	NA	NA	NA	NA
vha-12(ok821)	5	-	-	-	-	-	-	-	-	-	-	NA	NA	NA	NA
crt-1(ok948)	4	-	-	-	< 0.0001	0.0448	-	-	-	-	-	NA	NA	NA	NA
unc-32(e189) lin-12(n676n930); unc-42(e270) sel-11(ar39)	4	-	-	-	-	-	-	-	-	-	-	-	-	-	-
unc-32(e189) lin-12(n676n930); sqt-3(sc8) sel-1(e1948)	4	-	-	-	-	-	-	-	-	-	-	NA	NA	NA	NA
pek-1(ok275)	4	-	-	-	-	-	-	-	-	-	-	-	-	-	-
hsp-4::GFP(zcIs4)	5	-	-	-	-	-	-	-	-	-	-	-	-	-	-
ire-1(v33)	5	-	-	-	-	-	0.0098	0.0006	0.0006	-	-	NA	NA	NA	NA
ire-1(zc14);hsp-4::GFP(zcIs4)	5	-	-	-	-	0.0407	-	-	-	-	-	NA	NA	NA	NA
xbp-1(zc12);hsp-4::GFP(zcIs4)	9	0.038	-	0.0282	0.0351	-	-	-	-	-	-	-	-	-	NA

(b)

Adjusted P values for Dunnett multiple comparison with wild type (2-way ANOVA)	number of independent experiments	day 1	day 2	day 3	day 4	day 5	day 6	day 7	day 8	day 9	day 10	day 11	day 12	day 13	day 14
daf-16(mgDf50)	9	-	< 0.0001	< 0.0001	< 0.0001	< 0.0001	< 0.0001	< 0.0001	< 0.0001	0.0093	0.0028	NA	NA	NA	NA
daf-16(mgDf50);daf-2(e1370)	5	-	-	-	-	0.0129	0.0099	0.0062	0.0076	-	-	NA	NA	NA	NA
daf-16(mgDf50);daf-2(m577)	5	0.0268	< 0.0001	-	-	-	-	-	-	-	-	NA	NA	NA	NA
daf-2(e1370)	11	< 0.0001	-	0.0032	0.0005	0.0317	-	-	-	-	-	-	-	-	-
daf-2(m577)	7	0.0024	0.0492	-	-	-	-	-	-	-	-	-	-	-	-
eat-2(ad465)	5	-	0.001	0.017		0.025	0.0304	0.0006	0.0001	< 0.0001	0.0342	NA	NA	NA	NA
skn-1(zu67)	9	-	-	-	-	-	0.0183	0.001	< 0.0001	0.2095	NA	NA	NA	NA	NA
SKN-1::GFP	3	0.0298	0.0202	0.0071							NA	NA	NA	NA	NA
hsf-1(sy441)	8	-	-	-	-	-	-	-	-	0.0005	< 0.0001	< 0.0001	NA	NA	NA
atg-2(bp576)	15	0.0241	< 0.0001	0.0004	-	-	-	-	-	0.0009	< 0.0001	0.0076	< 0.0001	NA	NA
atg-4.1(bp501)	15	0.0052	0.008	-	-	-	-	-	-	-	-	-	-	NA	NA
atg-13(bp414)	15	< 0.0001	0.0014	0.0046	-	-	-	-	0.0035	< 0.0001	< 0.0001	NA	NA	NA	NA
atg-18(gk378)	15	0.0011	0.021	0.0004	0.0226	-	-	0.0082	< 0.0001	< 0.0001	0.0018	NA	NA	NA	NA
hif-1(ia4)	5	-	-	-	-	-	-	-	-	-	-	-	NA	NA	NA
aak-2(ok584)	5	-	-	0.0286	-	-	-	-	-	-	NA	NA	NA	NA	NA
vha-12(ok821)	5	-	-	-	-	-	-	-	0.0176	0.0429	-	NA	NA	NA	NA

Table S1 | Day-by-day comparison of age-matched mutants with wild-type control animals for 42°C heat stress resistance (a) and 7% *t*-BHP severe oxidative stress resistance (b). NA: not applicable (not enough worms survived, unusable curves, or untested condition).

(a)

Adjusted P values for Dunnett multiple comparison with age (2-way ANOVA)	number of independent experiments	comparison to day 1												
		day 2	day 3	day 4	day 5	day 6	day 7	day 8	day 9	day 10	day 11	day 12	day 13	day 14
wild type	27	-	0.0027	0.0067	0.0004	< 0.0001	< 0.0001	< 0.0001	-	-	-	-	-	-
daf-16(mgDf50)	5	-	-	-	-	-	-	-	-	NA	NA	NA	NA	NA
daf-16(mgDf50);daf-2(e1370)	5	-	-	-	-	-	-	-	-	-	NA	NA	NA	NA
daf-16(mgDf50);daf-2(m577)	5	-	-	-	-	-	0.0207	-	-	NA	NA	NA	NA	NA
daf-2(e1370)	9	-	0.0179	0.0006	< 0.0001	-	0.0245	0.0010	0.0081	0.0589	0.0490	< 0.0001	-	-
daf-2(m577)	5	-	0.0062	0.0049	0.0199	0.0011	-	-	-	0.0078	0.0171	-	-	-
daf-2(e1368)	4	-	-	-	-	-	0.0406	0.0200	0.0014	0.0339	0.0062	NA	NA	NA
eat-2(ad465)	5	-	0.0023	-	0.0215	0.0256	0.0033	0.0391	-	0.0010	-	NA	NA	NA
eat-20(nc4)	5	-	-	-					-	0.0009	-	NA	NA	NA
phm-2(ad597)	5	-	-	-	-	-	-	-	0.0149	0.0271	-	NA	NA	NA
skn-1(zu67)	5	-	-	-	-	-	-	-	-	-	NA	NA	NA	NA
hsf-1(sy441)	4	-	-	-	-	-	-	-	-	-	-	NA	NA	NA
hsp-16.2(gk249)	4	-	-	-	-	-	-	-	-	-	-	NA	NA	NA
atg-2(bp576)	14	-	-	-	-	-	-	-	-	-	-	-	NA	NA
atg-3(bp412)	9	-	-	-	-	-	-	-	-	-	-	-	NA	NA
atg-4.1(bp501)	14	-	-	-	< 0.0001	0.0002	< 0.0001	< 0.0001	< 0.0001	< 0.0001	0.0002	-	NA	NA
atg-7(bp411)	9	-	-	-	-	-	-	-	-	-	NA	NA	NA	NA
atg-9(bp564)	9	-	-	-	-	-	-	-	-	-	-	-	NA	NA
atg-13(bp414)	14	-	-	-	-	-	-	-	-	-	-	-	NA	NA
atg-18(gk378)	9	-	-	-	-	-	-	-	-	0.0102	-	-	NA	NA
hif-1(ia4)	5	-	-	-	-	-	0.0098	0.0120	-	-	-	-	NA	NA
aak-2(ok584)	5	-	-	-	-	-	-	-	-	NA	NA	NA	NA	NA
vha-12(ok821)	5	-	0.0041	0.0065	0.0006	0.0006	0.0504	0.0424	-	-	NA	NA	NA	NA
crt-1(ok948)	4	-	-	< 0.0001	0.0103	0.0219	0.0238	-	-	-	NA	NA	NA	NA
unc-32(e189) lin-12(n676n930); unc-42(e270) sel-11(ar39)	4	-	-	-	-	-	-	-	-	-	NA	NA	NA	NA
unc-32(e189) lin-12(n676n930); sqt-3(sc8) sel-1(e1948)	4	-	-	-	0.0406	< 0.0001	-	-	-	-	NA	NA	NA	NA
pek-1(ok275)	4	-	-	-	-		< 0.0001	< 0.0001	0.0002	0.0003	0.4592	-	-	-
hsp-4::GFP(zcIs4)	5	-	-	-	-	0.0169	0.0468	0.0320	-	-	-	-	-	-
ire-1(v33)	5	-	-	-	-	< 0.0001	< 0.0001	0.0199	0.0003	-	NA	NA	NA	NA
ire-1(zc14);hsp-4::GFP(zcIs4)	5	-	-	0.0474	-	-	-	-	-	-	NA	NA	NA	NA
xbp-1(zc12);hsp-4::GFP(zcIs4)	9	-	-	-	-	-	-	-	-	-	-	-	-	NA

(b)

Adjusted P values for Dunnett multiple comparison with age (2-way ANOVA)	number of independent experiments	comparison to day 2											
		day 3	day 4	day 5	day 6	day 7	day 8	day 9	day 10	day 11	day 12	day 13	day 14
wild type	27	-	-	-	0.0009	0.0256	0.0211	-	-	-	-	-	-
daf-16(mgDf50)	5	-	-	-	-	-	-	-	NA	NA	NA	NA	NA
daf-16(mgDf50);daf-2(e1370)	5	-	-	-	-	-	-	-	-	NA	NA	NA	NA
daf-16(mgDf50);daf-2(m577)	5	-	-	-	-	0.0150	-	-	NA	NA	NA	NA	NA
daf-2(e1370)	9	-	0.0032	< 0.0001	-	-	-	0.0340	-	-	0.0003	-	-
daf-2(m577)	5	-	-	-	-	-	-	-	-	-	-	-	-
daf-2(e1368)	4	-	-	-	-	-	0.0228	0.0166	0.0375	-	NA	NA	NA
eat-2(ad465)	5	-	-	-	-	-	-	-	-	-	NA	NA	NA
eat-20(nc4)	5	-	-	-	-	-	-	-	-	-	NA	NA	NA
phm-2(ad597)	5	-	-	-	-	0.0196	0.0051	0.0004	0.0009	0.0122	NA	NA	NA
skn-1(zu67)	5	-	-	-	-	-	-	-	-	NA	NA	NA	NA
hsf-1(sy441)	4	-	-	-	-	-	-	-	-	-	NA	NA	NA
hsp-16.2(gk249)	4	-	-	-	-	-	-	-	-	-	NA	NA	NA
atg-2(bp576)	14	-	-	-	-	-	-	-	-	-	-	NA	NA
atg-3(bp412)	9	-	-	-	-	0.0256	-	0.0134	-	-	-	NA	NA
atg-4.1(bp501)	14	-	-	0.0015	0.0020	0.0011	0.0024	0.0005	0.0005	0.0011	-	NA	NA
atg-7(bp411)	9	-	-	-	-	-	-	-	-	NA	NA	NA	NA
atg-9(bp564)	9	-	-	-	-	-	-	-	-	-	-	NA	NA
atg-13(bp414)	14	-	-	-	-	-	-	-	-	-	-	NA	NA
atg-18(gk378)	9	-	-	-	-	-	-	-	-	-	-	NA	NA
hif-1(ia4)	5	-	-	-	-	0.0415	0.0465	-	-	-	-	NA	NA
aak-2(ok584)	5	-	-	-	-	-	-	-	NA	NA	NA	NA	NA
vha-12(ok821)	5	-	-	-	-	-	-	-	-	NA	NA	NA	NA
crt-1(ok948)	4	-	0.0015	0.0041	0.0089	0.0098	-	-	-	NA	NA	NA	NA
unc-32(e189) lin-12(n676n930); unc-42(e270) sel-11(ar39)	4	-	-	-	-	-	-	-	-	NA	NA	NA	NA
unc-32(e189) lin-12(n676n930); sqt-3(sc8) sel-1(e1948)	4	-	-	-	0.0001	-	-	-	-	NA	NA	NA	NA
pek-1(ok275)	4	-	-	-	-	0.0035	0.0166	0.0007	0.0304	-	-	-	-
hsp-4::GFP(zcIs4)	5	-	-	-	-	-	-	-	-	-	-	-	-
ire-1(v33)	5	-	-	0.0051	0.0027	0.0009	-	0.0227	-	NA	NA	NA	NA
ire-1(zc14);hsp-4::GFP(zcIs4)	5	-	-	-	-	-	-	-	-	NA	NA	NA	NA
xbp-1(zc12);hsp-4::GFP(zcIs4)	9	-	-	-	-	-	-	-	-	-	-	-	NA

(c)

Adjusted P values for Dunnett multiple comparison with age (2-way ANOVA)	number of independent experiments	comparison to day 3										
		day 4	day 5	day 6	day 7	day 8	day 9	day 10	day 11	day 12	day 13	day 14
wild type	27	-	-	-	-	-	-	-	-	-	-	-
daf-16(mgDf50)	5	-	-	-	-	-	-	NA	NA	NA	NA	NA
daf-16(mgDf50);daf-2(e1370)	5	-	-	-	-	-	-		NA	NA	NA	NA
daf-16(mgDf50);daf-2(m577)	5	-	-	-	-	-	-	NA	NA	NA	NA	NA
daf-2(e1370)	9	-	0.0106	-	-	-	-	-	-	-	-	-
daf-2(m577)	5	-	-	-	-	-	-	-	-	-	-	-
daf-2(e1368)	4	-	-	-	-	-	-	-	-	NA	NA	NA
eat-2(ad465)	5	-	-	-	-	-	-	0.0362	-	NA	NA	NA
eat-20(nc4)	5	-	-	-	-	-	-	-	-	NA	NA	NA
phm-2(ad597)	5	-	-	-	0.0072	0.0009	< 0.0001	0.0003	-	NA	NA	NA
skn-1(zu67)	5	-	-	-	-	-	-	-	NA	NA	NA	NA
hsf-1(sy441)	4	-	-	-	-	-	-	-	-	NA	NA	NA
hsp-16.2(gk249)	4	-	-	-	-	-	-	-	-	NA	NA	NA
atg-2(bp576)	14	-	-	-	-	-	-	-	-	-	NA	NA
atg-3(bp412)	9	-	-	-	-	-	0.0288	-	-	-	NA	NA
atg-4.1(bp501)	14	-	0.0139	0.0200	0.0108	0.0224	0.0048	0.0065	0.0114	-	NA	NA
atg-7(bp411)	9	-	-	-	-	-	-	-	NA	NA	NA	NA
atg-9(bp564)	9	-	-	-	-	-	-	-	-	-	NA	NA
atg-13(bp414)	14	-	-	-	-	-	-	-	-	-	NA	NA
atg-18(gk378)	9	-	-	-	-	-	-	-	-	-	NA	NA
hif-1(ia4)	5	-	-	-	0.0415	0.0346	-	-	-	-	NA	NA
aak-2(ok584)	5	-	-	-	-	-	-	NA	NA	NA	NA	NA
vha-12(ok821)	5	-	-	-	-	-	0.0119	0.0006	NA	NA	NA	NA
crt-1(ok948)	4	-	-	-	-	-	-	-	NA	NA	NA	NA
unc-32(e189) lin-12(n676n930); unc-42(e270) sel-11(ar39)	4	-	-	-	-	-	-	-	NA	NA	NA	NA
unc-32(e189) lin-12(n676n930); sqt-3(sc8) sel-1(e1948)	4	0.0130	0.0099	< 0.0001	-	-	-	-	NA	NA	NA	NA
pek-1(ok275)	4	-	-	-	0.0067	0.0337	0.0141	-	-	-	-	-
hsp-4::GFP(zcIs4)	5	-	-	-	-	-	-	-	-	-	-	-
ire-1(v33)	5			0.0015	0.0005	-	0.0208	-	NA	NA	NA	NA
ire-1(zc14);hsp-4::GFP(zcIs4)	5	-	-	-	-	-	-	-	NA	NA	NA	NA
xbp-1(zc12);hsp-4::GFP(zcIs4)	9	-	-	-	-	-	-	-	-	-	-	NA

Table S2 | Comparisons within genotype between days for 42°C heat stress resistance: comparison to day 1 (a), day 2 (b), and day 3 (c). NA: not applicable (not enough worms survived, unusable curves, or untested condition).

(a)

Adjusted P values for Dunnett multiple comparison with age (2-way ANOVA)	number of independent experiments	comparison to day 1												
		day 2	day 3	day 4	day 5	day 6	day 7	day 8	day 9	day 10	day 11	day 12	day 13	day 14
wild type	24	< 0.0001	< 0.0001	< 0.0001	< 0.0001	< 0.0001	< 0.0001	< 0.0001	< 0.0001	< 0.0001	< 0.0001	< 0.0001	< 0.0001	0.0009
daf-16(mgDf50)	9	-	0.0443	-	-	-	0.0002	< 0.0001	< 0.0001	0.0023	NA	NA	NA	NA
daf-16(mgDf50);daf-2(e1370)	5	-	-	-	-	-	-	0.0494	-	-	NA	NA	NA	NA
daf-16(mgDf50);daf-2(m577)	5	-	0.0001	< 0.0001	< 0.0001	< 0.0001	< 0.0001	< 0.0001	< 0.0001	< 0.0001	NA	NA	NA	NA
daf-2(e1370)	11	-	0.0001	< 0.0001	< 0.0001	0.0002	0.0025	0.0002	0.0032	< 0.0001	0.0011	0.0044	0.0001	0.0116
daf-2(m577)	7	-	0.0205	0.0006	0.0214	0.0004	0.0004	< 0.0001	< 0.0001	< 0.0001	0.0014	0.0023	0.0008	0.0213
eat-2(ad465)	5	-	0.0116	< 0.0001	< 0.0000	< 0.0001	0.0002	< 0.0001	0.0095	-	NA	NA	NA	NA
skn-1(zu67)	9	-	-	0.0133	0.0039	-	-	-	-	NA	NA	NA	NA	NA
SKN-1::GFP	3	-	0.0087	-	-	-	-	-	-	NA	NA	NA	NA	NA
hsf-1(sy441)	8	0.0475	-	-	-	0.0186	0.005	< 0.0001	< 0.0001	< 0.0001	< 0.0001	NA	NA	NA
atg-2(bp576)	15	< 0.0001	< 0.0001	< 0.0001	0.0011	< 0.0001	0.0007	0.0011	-	-	-	-	NA	NA
atg-4.1(bp501)	15	0.0048	-	0.0029	< 0.0001	< 0.0001	< 0.0001	< 0.0001	< 0.0001	< 0.0001	< 0.0001	0.0002	NA	NA
atg-13(bp414)	15	-	0.0045	0.0074	-	0.0139	-	-	-	-	NA	NA	NA	NA
atg-18(gk378)	15	0.0313	< 0.0001	< 0.0001	0.0108	0.0324	-	-	-	-	NA	NA	NA	NA
hif-1(ia4)	5	-	0.0081	< 0.0001	0.0010	0.0002	0.0022	0.0025	0.0068	-	-	NA	NA	NA
aak-2(ok584)	5	0.0116	0.0061	< 0.0001	< 0.0001	< 0.0001	< 0.0001	< 0.0001	< 0.0001	NA	NA	NA	NA	NA
vha-12(ok821)	5	0.0252	< 0.0001	< 0.0001	< 0.0001	< 0.0001	< 0.0001	< 0.0001	0.0023	0.0086	NA	NA	NA	NA

(b)

Adjusted P values for Dunnett multiple comparison with age (2-way ANOVA)	number of independent experiments	comparison to day 2											
		day 3	day 4	day 5	day 6	day 7	day 8	day 9	day 10	day 11	day 12	day 13	day 14
wild type	24	-	0.0263	0.0005	< 0.0001	< 0.0001	< 0.0001	< 0.0001	< 0.0001	< 0.0001	< 0.0001	-	-
daf-16(mgDf50)	9	-	-	-	-	0.0057	< 0.0001	< 0.0001	0.0149	NA	NA	NA	NA
daf-16(mgDf50);daf-2(e1370)	5	-	-	-	-	-	-	-	-	NA	NA	NA	NA
daf-16(mgDf50);daf-2(m577)	5	0.044	< 0.0001	< 0.0001	< 0.0001	< 0.0001	< 0.0001	< 0.0001	0.0033	NA	NA	NA	NA
daf-2(e1370)	11	-	0.0009	0.0057	0.0102	-	0.0132	-	0.0054	0.0363	-	0.0058	-
daf-2(m577)	7	-	-	-	-	-	0.0091	0.0076	0.0117	-	-	-	-
eat-2(ad465)	5	-	0.0213	0.0407	0.0067	0.0551	0.0165	-	-	NA	NA	NA	NA
skn-1(zu67)	9	-	-	0.0496	-	-	-	-	NA	NA	NA	NA	NA
SKN-1::GFP	3	-	-	-	-	-	-	-	NA	NA	NA	NA	NA
hsf-1(sy441)	8	-	-	-	-	-	0.0033	< 0.0001	< 0.0001	< 0.0001	NA	NA	NA
atg-2(bp576)	15	-	-	-	-	-	-	0.0117	0.0247	-	0.0027	NA	NA
atg-4.1(bp501)	15	-	-	-	-	-	-	-	0.0208	0.0234	-	NA	NA
atg-13(bp414)	15	-	-	-	-	-	-	0.0289	-	NA	NA	NA	NA
atg-18(gk378)	15	-	-	-	-	-	0.0185	0.0014	-	NA	NA	NA	NA
hif-1(ia4)	5	-	-	-	-	-	-	-	-	-	NA	NA	NA
aak-2(ok584)	5	-	-	-	0.0106	0.0088	0.0014	0.0002	0.019	NA	NA	NA	NA
vha-12(ok821)	5	-	-	0.0088	-	-	-	-	-	NA	NA	NA	NA

(c)

Adjusted P values for Dunnett multiple comparison with age (2-way ANOVA)	number of independent experiments	comparison to day 3										
		day 4	day 5	day 6	day 7	day 8	day 9	day 10	day 11	day 12	day 13	day 14
wild type	24	-	-	0.0093	0.0034	< 0.0001	< 0.0001	< 0.0001	< 0.0001	0.0012	-	-
daf-16(mgDf50)	9	-	-	-	-	0.0137	0.0007	0.2156	NA	NA	NA	NA
daf-16(mgDf50);daf-2(e1370)	5	-	-	-	-	-	-	-	NA	NA	NA	NA
daf-16(mgDf50);daf-2(m577)	5	-	-	-	0.0376	0.0055	0.0085	-	NA	NA	NA	NA
daf-2(e1370)	11	-	-	-	-	-	-	-	-	-	-	-
daf-2(m577)	7	-	-	-	-	-	0.0457	-	-	-	-	-
eat-2(ad465)	5	-	-	-	-	-	-	-	NA	NA	NA	NA
skn-1(zu67)	9	-	-	-	-	-	-	NA	NA	NA	NA	NA
SKN-1::GFP	3	-	-	-	-	-	-	NA	NA	NA	NA	NA
hsf-1(sy441)	8	-	-	-	-	-	0.0034	< 0.0001	< 0.0001	NA	NA	NA
atg-2(bp576)	15	-	-	-	-	-	0.0008	0.0018	0.0450	0.0004	NA	NA
atg-4.1(bp501)	15	-	-	-	-	-	0.0114	< 0.0001	< 0.0001	-	NA	NA
atg-13(bp414)	15	-	-	-	-	0.0199	0.0008	0.0027	NA	NA	NA	NA
atg-18(gk378)	15	-	-	0.0195	0.0001	< 0.0001	< 0.0001	0.0107	NA	NA	NA	NA
hif-1(ia4)	5	-	-	-	-	-	-	-	-	NA	NA	NA
aak-2(ok584)	5		0.0196	0.0165	0.0027	0.0004	0.0295	NA	NA	NA	NA	NA
vha-12(ok821)	5	-	-	-	-	-	-	-	NA	NA	NA	NA

Table S3 | Comparisons within genotype between days for 7% *t*-BHP severe oxidative stress resistance: comparison to day 1 (a), day 2 (b), and day 3 (c). NA: not applicable (not enough worms survived, unusable curves, or untested condition).

(a)

Adjusted P values for Dunnett multiple comparison with age (2-way ANOVA)	number of independent experiments	comparison to day of maximum resistance (Rmax)													
		day 1	day 2	day 3	day 4	day 5	day 6	day 7	day 8	day 9	day 10	day 11	day 12	day 13	day 14
wild type	27	< 0.0001	0.0004	-	-	-	Rmax	-	-	0.0317	0.0281	0.0007	0.0072	-	-
daf-16(mgDf50)	5	-	-	Rmax	-	-	-	-	-	-	NA	NA	NA	NA	NA
daf-16(mgDf50);daf-2(e1370)	5	-	-	-	-	-	-	Rmax	-	-	-	NA	NA	NA	NA
daf-16(mgDf50);daf-2(m577)	5	0.0058	0.0042	-	-	-	-	Rmax	-	-	NA	NA	NA	NA	NA
daf-2(e1370)	9	< 0.0001	< 0.0001	0.0079	-	Rmax	0.0008	0.0032	0.0037	0.0153	0.0015	-	-	-	-
daf-2(m577)	5	0.0022	-	-	Rmax	-	-	-	-	-	-	-	-	-	-
daf-2(e1368)	4	0.0106	-	-	-	-	-	-	Rmax	-	-	-	NA	NA	NA
eat-2(ad465)	5	0.0012	-	Rmax	-	-	-	-	-	-	0.0379	NA	NA	NA	NA
eat-20(nc4)	5	0.0429	-	-	-	-	-	Rmax	-	-	NA	NA	NA	NA	NA
phm-2(ad597)	5	-	-	-	Rmax	-	-	0.0007	< 0.0001	< 0.0001	0.0001	0.0012	0.0047	NA	NA
skn-1(zu67)	5	-	-	-	Rmax	-	-	-	-	-	-	NA	NA	NA	NA
hsf-1(sy441)	4	Rmax	-	-	-	-	-	-	-	-	-	-	NA	NA	NA
hsp-16.2(gk249)	4	-	-	-	0.0413	-	0.0459	-	Rmax	-	-	-	NA	NA	NA
atg-2(bp576)	14	-	-	-	-	-	Rmax	-	-	-	-	-	-	NA	NA
atg-3(bp412)	9	-	-	-	-	-	Rmax	-	-	-	-	-	-	NA	NA
atg-4.1(bp501)	14	< 0.0001	0.0005	0.0080	-	-	-	Rmax	-	-	-	-	< 0.0001	NA	NA
atg-7(bp411)	9	-	-	-	-	-	-	-	-	Rmax	-	NA	NA	NA	NA
atg-9(bp564)	9	-	-	-	-	-	-	-	-	-	-	-	-	NA	NA
atg-13(bp414)	14	-	-	-	-	-	Rmax	-	-	-	-	-	-	-	NA
atg-18(gk378)	9	-	-	-	Rmax	-	-	-	-	-	0.0176	-	-	NA	NA
hif-1(ia4)	5	0.0060	0.0270	0.0270	0.0025	0.0343	-	-	Rmax	0.0267	0.0168	-	-	NA	NA
aak-2(ok584)	5	-	-	Rmax	-	-	-	-	0.0007	-	NA	NA	NA	NA	NA
vha-12(ok821)	5	0.0002	-	-	-	-	Rmax	-	-	0.0026	< 0.0001	NA	NA	NA	NA
crt-1(ok948)	4	0.0018	0.0007	-	Rmax	-	-	-	-	0.0416	0.0122	-	NA	NA	NA
unc-32(e189) lin-12(n676n930); unc-42(e270) sel-11(ar39)	4	-	-	-	-	-	-	Rmax	-	-	-	NA	NA	NA	NA
unc-32(e189) lin-12(n676n930); sqt-3(sc8) sel-1(e1948)	4	< 0.0001	< 0.0001	< 0.0001	-	-	Rmax	-	0.0066	-	< 0.0001	NA	NA	NA	NA
pek-1(ok275)	4	0.0005	0.0016	0.0334	0.0459	0.0424	-	Rmax	-	-	-	-	-	-	-
hsp-4::GFP(zcIs4)	5	0.0161	-	-	-	-	Rmax	-	-	-	-	0.0181	-	-	-
ire-1(v33)	5	< 0.0001	0.0003	0.0003	0.0009		-	Rmax	0.0410	-	-	NA	NA	NA	NA
ire-1(zc14);hsp-4::GFP(zcIs4)	5	0.0276	-	-	Rmax	-	-	-	-	-	-	NA	NA	NA	NA
xbp-1(zc12);hsp-4::GFP(zcIs4)	9	-	-	-	-	Rmax	-	-	-	-	-	0.0314	-	NA	NA

(b)

Adjusted P values for Dunnett multiple comparison with age (2-way ANOVA)	number of independent experiments	comparison to day of maximum resistance (Rmax)													
		day 1	day 2	day 3	day 4	day 5	day 6	day 7	day 8	day 9	day 10	day 11	day 12	day 13	day 14
wild type	24	< 0.0001	< 0.0001	< 0.0001	< 0.0001	< 0.0001	0.0014	0.0164	-	-	Rmax	-	-	-	-
daf-16(mgDf50)	9	< 0.0001	< 0.0001	0.0008	0.0002	0.0003	0.0003	-	-	Rmax	-	NA	NA	NA	NA
daf-16(mgDf50);daf-2(e1370)	5	-	-	-	Rmax	-	-	-	-	-	-	NA	NA	NA	NA
daf-16(mgDf50);daf-2(m577)	5	< 0.0001	< 0.0001	0.0064	-	-	-	-	Rmax	-	-	NA	NA	NA	NA
daf-2(e1370)	11	< 0.0001	0.0009	-	Rmax	-	-	-	-	-	-	-	-	-	-
daf-2(m577)	7	< 0.0001	0.0104	-	-	-	-	-	Rmax	-	-	-	-	-	-
eat-2(ad465)	5	< 0.0001	0.0077	-	-	-	Rmax	-	-	-	-	NA	NA	NA	NA
skn-1(zu67)	9	0.0039	0.0496	-	-	Rmax	-	-	-	-	NA	NA	NA	NA	NA
SKN-1::GFP	3	0.0087	-	Rmax	-	-	-	-	-	-	NA	NA	NA	NA	NA
hsf-1(sy441)	8	< 0.0001	< 0.0001	< 0.0001	< 0.0001	< 0.0001	< 0.0001	< 0.0001	< 0.0001	< 0.0001	Rmax	-	NA	NA	NA
atg-2(bp576)	15	< 0.0001	-	Rmax	-	-	-	-	-	0.0008	0.0018	-	0.0004	NA	NA
atg-4.1(bp501)	15	< 0.0001	0.0144	-	-	-	-	-	-	-	-	Rmax	-	NA	NA
atg-13(bp414)	15	0.0072	-	Rmax	-	-	-	-	0.0199	0.0008	0.0027	NA	NA	NA	NA
atg-18(gk378)	15	< 0.0001	-	Rmax	-	-	0.0195	0.0001	< 0.0001	< 0.0001	0.0107	NA	NA	NA	NA
hif-1(ia4)	5	< 0.0001	-	-	Rmax	-	-	-	-	-	-	-	NA	NA	NA
aak-2(ok584)	5	0.0061	-	Rmax	-	0.0196	0.0165	0.0027	0.0004	0.0295	NA	NA	NA	NA	NA
vha-12(ok821)	5	< 0.0001	0.0055	-	-	Rmax	-	-	-	-	-	NA	NA	NA	NA

Table S4 | Comparison within genotype to the day of maximum resistance (Rmax). NA: not applicable (not enough worms survived, unusable curves, or untested condition).

	median lifespan	mean lifespan	Minimum resistance to HS 42°C	Mean resistance to HS 42°C	Maximum resistance to HS 42°C	Minimum resistance to *t*-BHP	Mean resistance to *t*-BHP	Maximum resistance to *t*-BHP
daf-2(m577)	24.0	29.34	128.7	168.6	193.0	111.9	139.7	149.8
daf-16(mgDf50); daf-2(e1370)	9.0	10.65	83.0	99.8	119.5	96.0	111.3	120.0
skn-1(zu67)	8.5	-	114.0	130.8	150.6	102.0	112.8	120.6
eat-2(ad1116)	13.0	17.71	106.0	152.7	180.5	77.4	103.8	114.0
daf-2(e1370)	26.0	33.90	163.1	197.7	235.3	109.8	140.4	157.0
daf-16(mgDf50); daf-2(m577)	9.0	-	85.5	111.4	139.5	74.8	114.6	132.0
aak-2(ok584)	10.0	9.50	96.0	121.4	138.2	77.0	113.0	130.2
daf-16(mgDf50)	9.0	8.84	94.0	100.3	111.4	79.6	98.1	119.0
wild type	12.0	13.47	83.0	118.0	143.1	92.6	130.8	150.7
vha-12(ok821)	12.0	-	94.0	138.4	165.6	88.0	119.1	133.0
hif-1(ia4)	12.5	-	110.0	135.6	172.0	100.8	124.2	134.8
hsf-1(sy441)	9.0	8.22	75.0	106.4	128.0	107.6	145.2	218.0
atg-18(gk378)	8.5	10.21	56.0	86.4	108.0	107.0	122.0	142.7
atg-2()	11.0	12.13	36.0	92.0	114.2	99.0	124.9	142.7
atg-4.1()	11.0	11.49	40.0	113.0	137.8	110.0	136.8	154.0
atg-13()	10.5	12.17	56.0	83.7	96.2	115.3	126.9	140.4
phm-2(ad597)	15.0	14.85	86.0	124.3	176.7	88.0	115.3	136.0
wild type + 50mM metformin	17.0	17.26	71.0	118.2	170.0	77.0	109.4	139.0

Table S5 | Table of median (matching estimates for the LFASS stress assays performed) or mean lifespan values (combining experiments performed in our lab and in the Cabreiro lab using the same growth conditions) (Cabreiro et al., 2013; Riesen et al., 2014; Tullet et al., 2014), see Experimental Procedures, and Max, Min and Mean severe stress resistance measured over the first week of adulthood, used for the correlation plots in Figure 3c-d. Resistance levels are expressed in min, corresponding to the median time of death in LFASS assays.
